# De Novo Discovery of Bioactive Cyclic Peptides via Cellular Selection of Genetically Encoded Libraries

**DOI:** 10.1002/cbic.70532

**Published:** 2026-09-23

**Authors:** Linwei Yang, Abhijit Behera, Hannah J. Meyers, Tina Wang

**Affiliations:** ^1^ Department of Chemistry University of Wisconsin‐Madison Madison Wisconsin USA; ^2^ Department of Biochemistry University of Wisconsin‐Madison Madison Wisconsin USA

**Keywords:** cyclic peptide, genetic selections, peptides

## Abstract

Cellular selection has emerged as a powerful approach for high throughput de novo discovery of bioactive cyclic peptides (CPs). This strategy coexpresses genetically encoded CP libraries with the target of interest in cells and uses customizable selection systems to link desired CP activities to a selectable phenotype to directly identify functional inhibitors. Cellular selections have yielded many CP inhibitors against a diverse range of therapeutic targets and have been continuously empowered by expansions in the CP diversity and selection repertoire. In this review, we summarize studies on cellular selection using genetically encoded CP libraries and discuss opportunities for future application of this technology.

## Graphical abstract

Genetically encoded cyclic peptide libraries can be employed in cellular selection platforms to identify cyclic peptide inhibitors for therapeutic targets by directly selecting for desired biological activities. This review systematically summarizes representative studies and suggests future opportunities for using cellular selection for de novo cyclic peptide discovery.

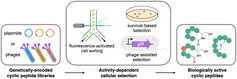

## 1 Introduction

Cyclic peptides (CPs) represent a versatile class of molecular scaffolds for drug discovery due to their modular and genetically encodable composition, which enables the generation of highly diverse libraries through synthetic and molecular biology methods (or a combination of both) [1, 2, 3, 4]. Display‐based platforms, such as phage and mRNA display, have facilitated the de novo identification of CP lead compounds from large, genetically encoded libraries [5, 6]. Platforms like random nonstandard peptides integrated discovery can generate extremely large CP libraries (>10^12^ variants) incorporating noncanonical amino acids (ncAAs) and unnatural backbones to identify novel ligands against diverse targets [7]. However, these methods typically rely on binding‐based selections, which may enrich high‐affinity binders that lack functional biological activity. Furthermore, display‐based approaches are generally restricted to targets that can be purified and immobilized, limiting their application for proteins that are poorly expressed, intrinsically disordered, or aggregation‐prone.

In contrast, cellular selections enable the direct identification of bioactive CPs by coupling their function to a selectable phenotype within a host cell. In this approach, CPs are biosynthesized intracellularly, and their desired activity, such as target inhibition, is linked to a reporter output like cell survival, fluorescence, or phage propagation. Because targets are expressed in an intracellular environment, this strategy also bypasses the need for protein purification and expands the scope of accessible targets. In addition to head‐to‐tail cyclized peptides, these systems have been used to investigate CPs containing ncAAs and sidechain‐to‐sidechain crosslinks [8, 9, 10, 11, 12]. Cellular selection has successfully identified bioactive CPs for challenging targets, including protein–protein interactions (PPIs) and aggregation‐prone proteins, and thus serves as an interesting complementary approach to display‐based methods for de novo CP discovery [13, 14, 15, 16, 17, 18, 19, 20, 21, 22, 23, 24].

In this review, we perform a survey of studies on the cellular selection of genetically encoded CP libraries (Table S1). We summarize representative works with a focus on CP scaffolds, targets of interest, selection systems, and outcomes. The discussion is organized by CP scaffold and further categorized by target class and selection mechanism. Finally, we discuss current limitations and future opportunities for the development of cellular selection platforms to accelerate the de novo discovery of potent bioactive CPs.

## 2 Split Intein‐Mediated Circular Ligation of Peptide and Proteins (SICLOPPS)

Arguably, the most widely used strategy for biosynthesizing cyclic peptides for cellular selections is the SICLOPPS method, introduced by Benkovic and colleagues in 1999 [25]. In the first report of the SICLOPPS method, Scott et al. demonstrated the biosynthesis of a cyclic octapeptide inhibitor of *Streptomyces antibioticus* tyrosinase in *Escherichia coli.* In the years since this pioneering study, SICLOPPS has been used to generate diverse genetically encoded CP libraries that can be selected using a variety of hosts, including yeast and mammalian cells [9, 16, 26, 27, 28]. In this section, we provide a general introduction to the mechanism of CP biosynthesis by SICLOPPS, followed by discussions of studies where SICLOPPS libraries were used to identify inhibitors of different classes of targets through genetic or phenotypic selections.

### 2.1 Biosynthesis of CPs Using SICLOPPS

**FIGURE 1:**
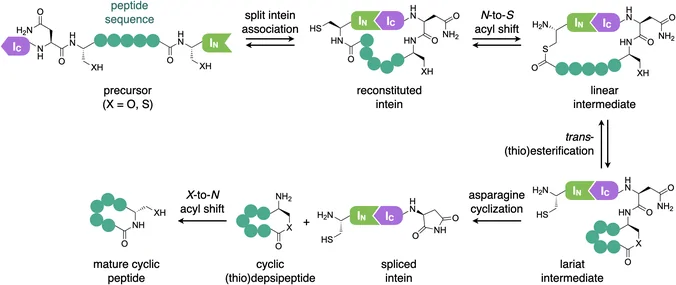
Production of head‐to‐tail cyclic peptides by SICLOPPS.

The SICLOPPS method uses intein splicing to generate cyclic peptides from a ribosomally synthesized precursor protein (Figure 1). Inteins are auto‐processing domains internally embedded in a precursor protein sequence that can remove themselves to covalently link their N‐ and C‐terminally flanking segments (also known as “exteins”) with a peptide bond in a process known as protein splicing [29]. Split inteins are a specific class of intein that exist as two separate fragments bearing the N‐terminal and C‐terminal halves of a full‐length intein (I_N_ and I_C_, respectively). By employing a fusion of a to‐be‐cyclized peptide sequence *in cis* between I_C_ and I_N_ of a split intein, SICLOPPS achieves the head‐to‐tail cyclization of a target peptide upon intein splicing (Figure 1).

The majority of studies employing SICLOPPS use the DnaE split intein from *Synechocystis* sp. PCC6803 (Ssp) to biosynthesize libraries of cyclic peptides ranging from 5 to 9 residues in length [30, 31]. The first cyclic peptide residue (corresponding to the +1 position in the C‐terminal extein) in the SICLOPPS precursor is fixed to cysteine or serine (or, less commonly, threonine) as a thiol or hydroxyl nucleophile at this position is required for the intein splicing mechanism. However, Ssp intein splicing can also be strongly influenced by the amino acids at the +2 and +3 positions of the C‐terminal extein, leading some SICLOPPS cyclic peptide sequences to undergo efficient cyclization and others to stall at various upstream steps [32].

A few studies have examined cyclization efficiencies of Ssp SICLOPPS‐encoded cyclic peptide libraries in a cellular context. Kritzer et al. randomly sampled 20 yeast cells transformed with a CX_7_ library (X = any canonical amino acid) and measured cyclization efficiency via Western blot analysis after a 5‐h expression [16]. The authors found that 4 clones contained frameshift or nonsense mutations, and one showed no splicing. All remaining clones exhibited splicing, albeit at varying efficiencies. Using a similar experimental procedure, Birkmose et al. observed comparable results where 87% of yeast cells carrying correctly encoded members of a CX_5_ library showed splicing, again with variable efficiencies [33]. Finally, Delivoria et al. analyzed 150 random clones from a CX_6_ library expressed in *E. coli* and found 45% corresponded to the correct construct and displayed splicing [20]. However, the abundance of the SICLOPPS precursor largely outweighed that of the spliced products, suggesting that many Ssp SICLOPPS sequences undergo incomplete splicing when expressed in cells. Although phenotypic activity detected in cells can arise from splicing intermediates rather than the mature CP, the success of cellular selections using SICLOPPS suggest that this complication does not prevent the ability of SICLOPPS to produce hits that are active as cyclic peptides. Indeed, we recently showed that SICLOPPS‐derived CPs were active when chemically synthesized and tested in vitro*,* even when their nonsplicing variants also displayed activity in a cellular context [22].

One strategy to improve CP production by SICLOPPS is to use different split inteins with increased splicing kinetics or better extein sequence tolerance. For instance, compared with the Ssp intein, the DnaE intein from *Nostoc punctiforme* (Npu) exhibits both faster splicing and greater sequence promiscuity [34, 35]. Townend and Tavassoli observed that employing the Npu intein in place of Ssp intein significantly improved splicing of a CX_5_ (X = any canonical amino acid) SICLOPPS library in *E. coli*, based on Western blot analysis of the bulk library [36]. However, the Npu SICLOPPS construct was observed to exert significant toxicity. To counteract this effect and to promote the selection of only completely spliced CP sequences, the authors fused a C‐terminal degron tag to the Npu SICLOPPS construct to mediate degradation of the full‐length precursor and any splicing intermediates. This successfully reduced Npu SICLOPPS‐associated toxicity and enabled “traceless” CP production in cells. Despite this improved performance, the application of the Npu SICLOPPS construct to a cellular selection of a CP library has not yet been reported.

### 2.2 Cellular Selections Using SICLOPPS Cyclic Peptide Libraries

In cellular selections, desired cyclic peptide activity (ex. target inhibition) is converted into a detectable readout (e.g., cell survival, fluorescence signal, and phage propagation) using a phenotypic or genetic selection system. Phenotypic systems are convenient due to their simplicity; however, customized biosensors and genetic circuits are usually necessary to accommodate targets that lack an inherently selectable phenotype. These genetic selection systems are often developed to fit different types of targets (e.g., PPIs, aggregation‐prone proteins, and proteases). Here, we discuss different targets of interest together with their compatible selection systems to summarize representative studies on cellular selection of CPs using SICLOPPS libraries. For a more comprehensive list of studies to date, please refer to Table S1.

#### 2.2.1 Selecting for PPI Disruptors Using the Reverse Two‐Hybrid System

**FIGURE 2:**
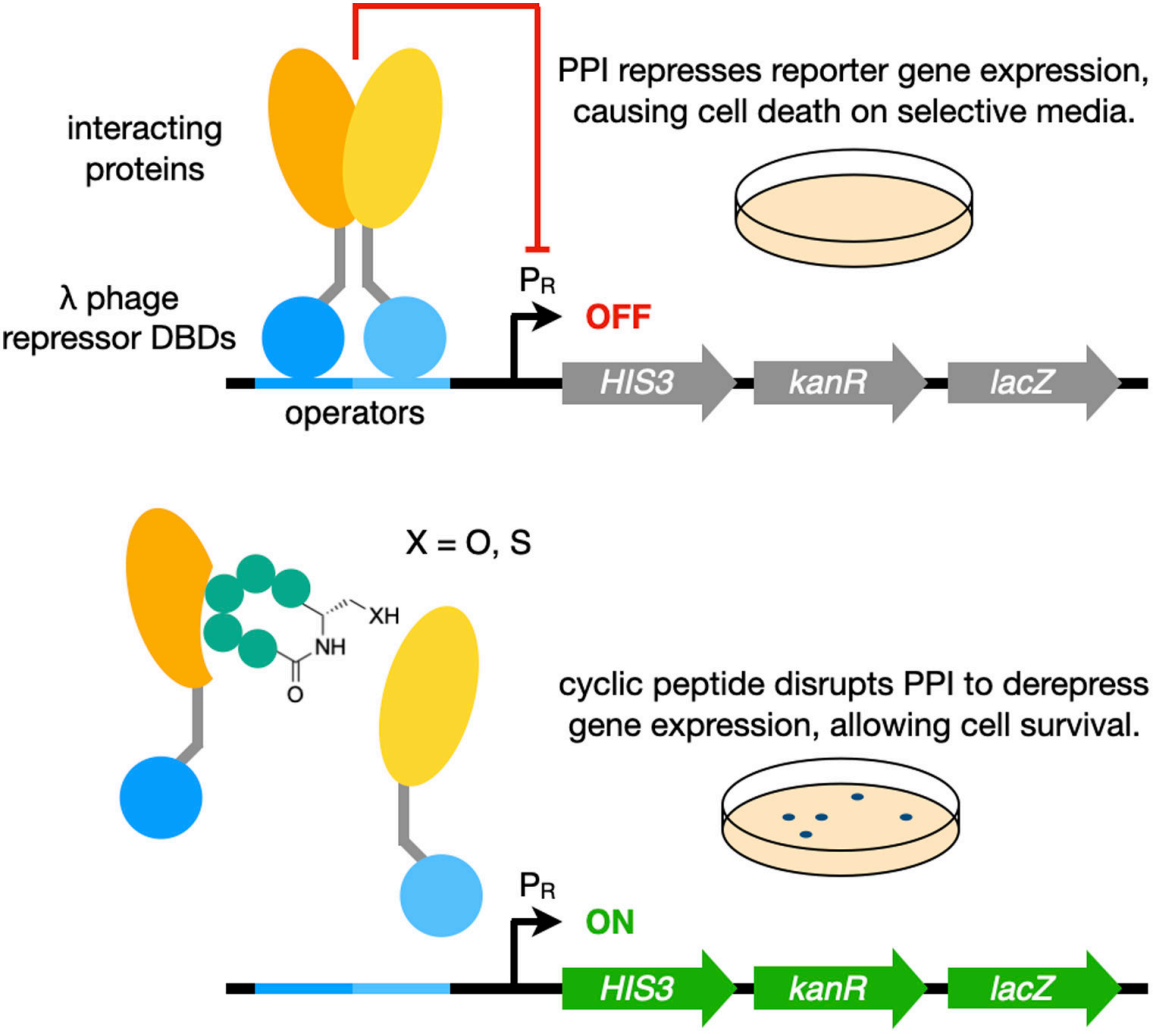
Overview of the reverse two‐hybrid system (R2H) for identifying protein–protein interaction disruptors.

PPIs are central to the regulation of biological processes and represent targets of high therapeutic interest. The reverse two‐hybrid (R2H) system has been employed extensively for the identification of PPI inhibitors using cellular selections. In the R2H (Figure 2), the target proteins involved in the PPI are fused to the DNA‐binding domains (DBDs) of a phage repressor in place of the phage repressor’s native dimerization domain. As dimerization of the DNA‐binding domain is required for phage repressor function, gene repression by the phage repressor becomes dependent on the PPI. A selection cassette containing reporter genes under the control of a promoter regulated by the phage repressor is then used to translate PPI dissociation into selectable phenotypes conferred by reporter gene expression [37]. In an early demonstration of using the R2H to identify PPI disruptors, Park and Raines selected a library of thioredoxin‐fused linear (non‐SICLOPPS) peptides in *E. coli* for dissociative inhibitors of the HIV protease homodimer using fusions of HIV protease to the λ phage repressor DBD [38]. Disruption of HIV protease dimerization led to derepression of a tetracycline resistance gene, enabling identification of inhibitors through antibiotic selection.

**FIGURE 3:**
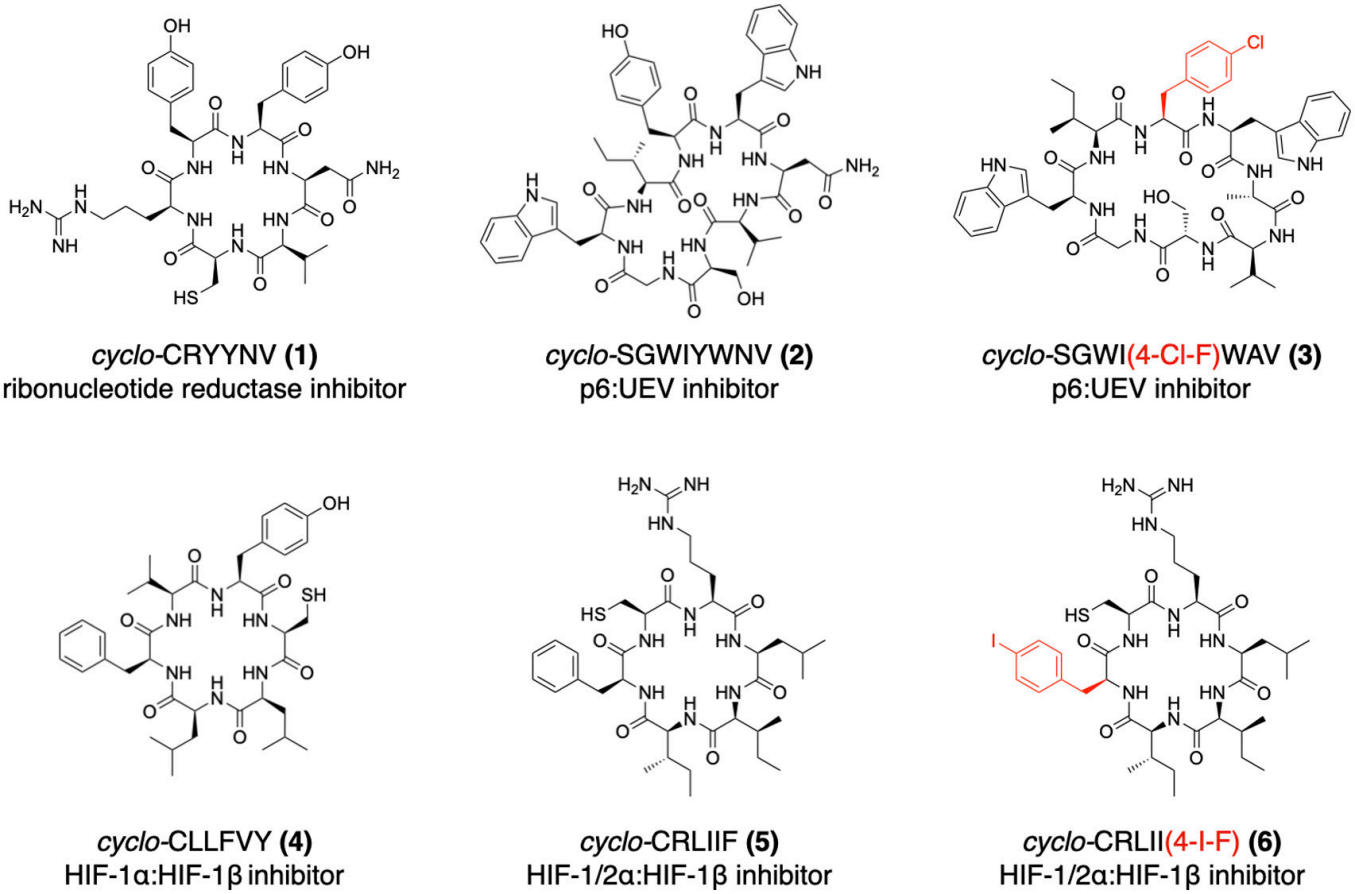
Representative cyclic peptide protein–protein interaction inhibitors isolated from R2H selections and their derivatives. ncAAs installed to improve hit potency are labeled in red.

To adapt the R2H to heterodimeric PPIs, Horswill et al. fused interacting proteins to chimeric phage repressor DBDs that recognize different operator sequences to repress a hybrid P_R_ promoter controlling expression of reporter genes HIS3, *kanR*, and *lacZ* [13, 39]. They applied this heterodimeric R2H to select CP disruptors of the ribonucleotide reductase complex from a CX_5_ Ssp SICLOPPS library (X = any canonical amino acid; ~10^8^ transformants) *in E. coli* by plating cells on selective media lacking histidine and containing kanamycin. Out of the 262 clones that passed the initial selection, only 24 showed activity when retransformed into fresh host cells, corresponding to a ~90% false‐positive rate in this survival‐based selection. These 24 hits were further narrowed down to eight top candidates by spot‐plating serial dilutions of bacteria expressing the SICLOPPS precursors on selective media. Among the eight final candidates, *cyclo*‐CRYYNV **(1)** (Figure 3) was identified as the most effective inhibitor in vitro, binding the ribonucleotide reductase small subunit with a K_d_ of ~53 µM (measured by tryptophan fluorescence quenching).

Since these early studies, the R2H has been applied to successfully identify CP inhibitors of other PPIs of therapeutic interest. For example, Tavassoli et al. targeted the interaction between the UEV domain of human TSG101 protein and the p6 subunit of the HIV Gag polyprotein, which is essential for HIV virus maturation, using an 8‐mer SICLOPPS library where the first three residues within the peptide sequence were fixed to SGW to promote Ssp intein splicing (~10^8^ transformants) [15]. After the initial selection followed by several rounds of secondary filtering (i.e., replating on selective agar and transforming into fresh host cells) to eliminate false positives, they isolated the sequence *cyclo‐*SGWIYWNV **(2)** (Figure 3), which also exhibited no activity against an off‐target PPI consisting of the ribonucleotide reductase complex. Chemically synthesized *cyclo‐*CGWIYWNV, fused to a Tat peptide via a disulfide bond to facilitate membrane penetration, inhibited Gag‐dependent virus‐like particle formation in 293T cells with an IC_50_ of ~7 µM. Unlike rationally designed peptide inhibitors of the p6:UEV interaction, *cyclo‐*SGWIYWNV bears no resemblance to the N‐terminal PTAP motif of p6, highlighting the ability of de novo discovery to identify new inhibitory motifs [40].

In a subsequent study, Lennard et al. tested chemically synthesized alanine‐scanned analogs of *cyclo‐*SGWIYWNV for p6:UEV inhibition using competitive ELISA and identified the tyrosine residue as essential for activity [41]. Guided by these results, the authors synthesized and tested analogs where the tyrosine phenol was replaced by other arenes, leading to the discovery of *cyclo‐*SGWI(4‐Cl‐F)WAV (4‐Cl‐F = 4‐chlorophenylalanine) **(3)** (Figure 3), which displayed ~10‐fold improved potency in inhibiting the p6:UEV interaction (IC_50_ ~ 5 µM by competitive ELISA) compared with *cyclo*‐SGWIYWNV. Notably, unlike *cyclo‐*SGWIYWNV, *cyclo‐*SGWI(4‐Cl‐F)WAV was found to be cell‐permeable and showed inhibition of virus‐like particle formation in 293T cells (IC_50_ of ~2 µM) without requiring a Tat peptide tag.

Another PPI targeted by several cellular selections using SICLOPPS libraries is the interaction between hypoxia‐inducible factors HIF‐1α and HIF‐1β, which plays a key role in the oncogenesis of various cancers [42]. Miranda et al. isolated the sequence *cyclo‐*CLLFVY **(4)** (Figure 3) from a CX_5_ library (~5 × 10^7^ transformants) using a R2H selection for HIF‐1α:HIF‐1β inhibitors [43]. Tat‐tagged *cyclo*‐CLLFVY was found to bind the PAS‐B domain of HIF‐1α with a K_d_ of 124 nM (measured by ITC) and inhibit the HIF‐1α:HIF‐1β interaction both in vitro (IC_50_ of 1.3 µM by competitive ELISA) and in cultured MCF‐7 and U2OS cells incubated under low‐O_2_ conditions. Inhibition of the HIF‐1‐mediated hypoxia response led to a decrease in HIF‐1‐regulated downstream effects, such as VEGF and carbonic anhydrase IX expression. Furthermore, *cyclo‐*CLLFVY was found to have no effect on the HIF‐2 response, which the authors hypothesized was due to structural differences between the HIF‐1α and HIF‐2α PAS‐B domains.

To identify inhibitors of both HIF‐1 and HIF‐2 activity, Ball et al. reapplied the CX_5_ SICLOPPS library in a two‐step selection procedure: first, the authors selected for HIF‐2α:HIF‐1β inhibitors, then subjected the CPs encoded by 125 surviving colonies to selection against the HIF‐1α:HIF‐1β interaction [44]. Three putative hits with highly similar sequences (CKLIIF, CRVIIF, and CRLIIF) were ultimately isolated. Of these, *cyclo*‐CRLIIF **(5)** (Figure 3), which bound both HIF‐1α and HIF‐2α (K_d_ = 3.8 µM and 10.9 µM, respectively), was selected for further investigation. Alanine scanning using chemically synthesized CPs identified the IFC motif as the pharmacophore responsible for HIF‐α engagement, prompting the authors to optimize *cyclo*‐CRLIIF by incorporating structurally similar noncanonical amino acids (ncAAs) into this tripeptide motif. These efforts ultimately yielded *cyclo*‐CRLII(4‐‍I‍‐‍F) (4‐I‐F = 4‐iodophenylalanine) **(6)** (Figure 3), which exhibited an improved *K*_d_ of 0.82 µM for HIF‐1α and 1.7 µM for HIF‐2α and inhibited both HIF‐1‐ and HIF‐2 dependent gene expression in cultured cancer cells.

To decrease bias and false positives in R2H selections, McDermott et al. recently replaced manual colony picking with next‐generation sequencing (NGS) of all surviving colonies [45]. Here, the authors reexamined selection for HIF‐1α:HIF‐1β inhibitors with the CX_5_ SICLOPPS library using a revised workflow involving bulk plasmid extraction from all postselection colonies, PCR amplification, and NGS analysis of CP sequences. After two rounds of this process, the authors identified ~1500 unique enriched CPs, which revealed several dominant sequence motifs, including CRX(L/V/F/Y)XL, which was not identified in prior selections. 13 out of the top 20 CPs, ranked by enrichment, showed appreciable binding (*K*_d_ < 100 µM) to the PAS‐B domain of HIF‐1α when chemically synthesized and assessed using MST. A newly identified sequence, *cyclo*‐CLLFCL, displayed the highest affinity to HIF‐1α (*K*_d_ ~ 0.47 µM) and strongly inhibited HIF‐1 activity in cells. However, the authors observed an imperfect correlation between sequence ranking and CP activity in vitro, with the most active sequence, *cyclo‐*CLLFCL, ranked as the lowest of the 20 CPs chosen for downstream testing. Even so, this study illustrates how the increased analytical throughput of NGS can be used to uncover previously overlooked sequences and to increase the positive hit rate of SICLOPPS selections.

In general, most CPs directly identified from R2H selections exhibit limited potency (typically ~0.1–100 µM *K*_d_ or IC_50_). By first identifying essential residues and then substituting them with ncAAs, some preliminary hits can be developed into more potent inhibitors, as demonstrated by the studies discussed above. However, the activity improvement gained by this approach has so far been limited to ~10‐fold, which may still be insufficient to meet the requirements for viable lead compounds. It is possible that CPs with higher activity reside in a broader chemical space not yet covered by the libraries assessed so far using the R2H selection, which have been modestly sized and limited to canonical amino acids. Moving forward, the examination of SICLOPPS libraries comprised of larger and more diverse CPs may enable the identification of more potent initial hits from R2H selections.

#### 2.2.2 Yeast Two‐Hybrid (Y2H) Selections for Target Protein Binders

**FIGURE 4:**
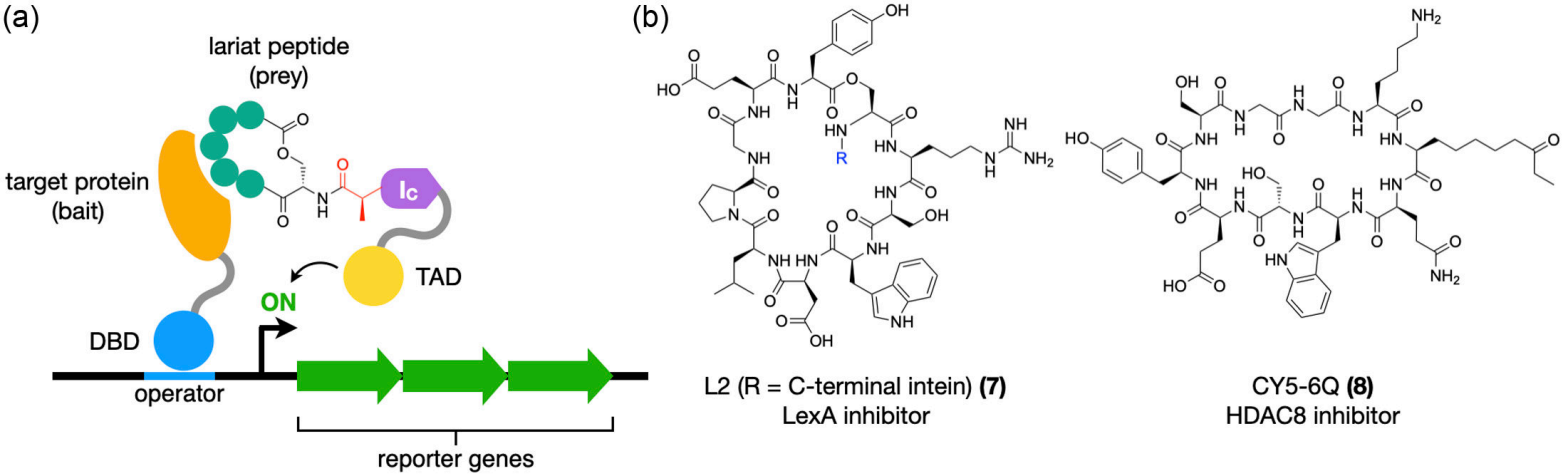
Yeast two‐hybrid (Y2H) system for identifying protein binders. (a) Overview of the Y2H system for selecting protein binders using fusions of SICLOPPS‐derived lariat peptides to a transcriptional activation domain (TAD). The alanine installed in place of asparagine in *I*_C_ to enable lariat peptide formation is labeled in red. (b) Representative protein binders isolated from Y2H selections.

The yeast two‐hybrid (Y2H) system, widely used for the study of protein–protein interactions [46], can also be applied to selecting genetically encoded CP libraries for binders to a target protein of interest [9, 27, 28]. In the Y2H strategy (Figure 4a), the target (“bait”) is localized to a specific promoter through fusion to a DBD, whereas the putative binder (“prey”) is fused to a transcriptional activation domain (TAD). Upon target:binder interaction, the TAD is recruited to the promoter to trigger the expression of downstream reporter genes (e.g., *HIS3*, *kanR*, and *lacZ*), allowing high‐throughput selection of binders. However, because the binder needs to be connected to a TAD, head‐to‐tail CPs are incompatible with the Y2H system due to the absence of free termini for fusion.

Barreto et al. adapted SICLOPPS for use with the Y2H system by installing a mutation in the SICLOPPS precursor to stall splicing at a lariat intermediate that retains the C‐terminal intein (I_C_), which can then be fused to a TAD [27]. The authors created a Y2H selection to identify binders to LexA, a central regulator of the SOS response in bacteria whose activation can lead to increased antibiotic resistance, by placing LexA operators upstream of a promoter controlling essential genes for leucine and purine biosynthesis and fusing a SICLOPPS lariat peptide library with a randomized loop region (SX_7_EY, ~5 × 10^6^ transformants) to a TAD. Out of 14 selected clones, 12 encoded the lariat peptide L2 **(7)** with a loop sequence of SRSWDLPGEY (with the side chain of the first serine engaged in a lactone). This sequence was determined to bind LexA in vitro with a K_d_ of ~37 µM. LC‐MS analysis of this SICLOPPS construct expressed in *E. coli* confirmed formation of **7**, although more than 50% of the species detected exhibited lactone hydrolysis. When expressed in *E. coli*, L2 was found to inhibit mitomycin C‐induced LexA autoproteolysis (measured by Western blot analysis), preserve LexA operator binding (measured by chromatin immunoprecipitation and qPCR), and sensitize the cells to mitomycin C treatment.

More recently, Kang et al. used the Y2H selection to identify binders of human histone deacetylase 8 (HDAC8) [9]. Here, the authors employed an engineered aminoacyl tRNA synthetase/tRNA pair to generate SICLOPPS lariat peptide libraries containing the ncAA 2‐amino‐8‐oxodecanoicacid (AODA) as a nonhydrolyzable structural mimic of HDAC8’s physiological substrate acetyllysine. Assessment of a TAD‐fused SX_3_θX_3_EY lariat library (X = any canonical amino acid, θ = AODA; ~10^6^ transformants) for *lacZ* reporter gene expression by blue/white colony screening, followed by testing of individual hits through a β‐galactosidase activity assay, yielded SGGLθLWSEY and SRFIθGWGEY. The authors then used a combination of alanine scanning and site‐saturation mutagenesis to further optimize these sequences to ultimately arrive at SGGLθQWSEY. When chemically resynthesized as an amide‐linked head‐to‐tail cyclized peptide, CY5‐6Q **(8)** was shown to bind HDAC8 with a K_d_ of 15.1 nM (measured by SPR) and inhibited the enzyme with an IC_50_ of 0.61 µM. In contrast, the original hit sequence SGGLθLWSEY exhibited a 10‐fold lower binding affinity for HDAC8 when tested in the same head‐to‐tail amide‐cyclized format. Molecular dynamics simulations predicted extension of AODA’s long aliphatic chain into the HDAC8 active site tunnel with the ketone coordinating the active site Zn^2+^. Notably, **8** is one of the most potent inhibitors identified through a cellular CP selection, highlighting how the strategic use of ncAAs combined with secondary screening can give rise to highly active sequences.

#### 2.2.3 Selecting for Protein Aggregation Suppressors

Aggregation‐prone proteins (APPs), particularly amyloidogenic proteins, have been implicated as molecular drivers of protein misfolding diseases. Despite their therapeutic importance as potential drug targets, de novo discovery of aggregation inhibitors remains challenging due to their aggregation‐prone nature, which complicates immobilization of purified protein targets in binding‐based screening or selection platforms. Recently, Ikenoue et al. reported the identification of CP inhibitors against α‐synuclein using mRNA display [47]; however, this strategy may be challenging to apply to APPs featuring faster aggregation kinetics, such as amyloid‐β (Aβ). In contrast, cellular selections for CP aggregation inhibitors, which can use phenotypic or biosensor‐based readouts of aggregation inhibition, have enabled the targeting of a broader range of APPs of therapeutic interest.

A widely used phenotypic readout of aggregation is the yeast synucleinopathy model, which operates through toxicity induced by human α‐synuclein expression. This model has been leveraged for fundamental studies on α‐synuclein misfolding as well as the engineering of factors that can alleviate its growth‐inhibitory effects [48]. In a foundational demonstration, Kritzer et al. employed the yeast synucleinopathy model to select a SICLOPPS CX_7_ library (X = any canonical amino acid, 5 × 10^6^ individual transformants) for members that would rescue cell viability [16]. Of 96 clones selected from colonies formed on selective agar inducing *GAL1* promoter‐controlled α‐synuclein expression, 31 demonstrated reproducible suppression of α‐synuclein toxicity when tested in fresh host cells. Furthermore, 2 of these 31 exhibited activities specific to α‐synuclein, leading to the identification of *cyclo‐*CPWCSTRV and *cyclo‐*CALCDPWW as initial hits. Alanine scanning of these two sequences identified a shared CXΦC sequence (X = any canonical amino acid, Φ = hydrophobic amino acid) as the active motif. In addition to alleviating α‐synuclein‐mediated cell death in yeast, both CPs were observed to reduce α‐synuclein‐dependent neurodegeneration when expressed in a *Caenorhabditis elegans* model.

The authors attempted to identify the cellular target(s) engaged by either CP through affinity enrichment experiments. However, although putative targets were enriched in these experiments, their overexpression or deletion failed to affect α‐synuclein toxicity or the ability of the CPs to suppress toxicity. Additionally, the CPs were found to have no effect on alleviating α‐synuclein‐dependent stalling of vesicle trafficking when expressed in cells, although they prevented the cell death that is caused by this stalling. Thus, this early study illustrates both the powerful ability of phenotypic selection to identify inhibitors with different and potentially new modes of action but also highlights challenges associated with false positives and target identification.

**FIGURE 5:**
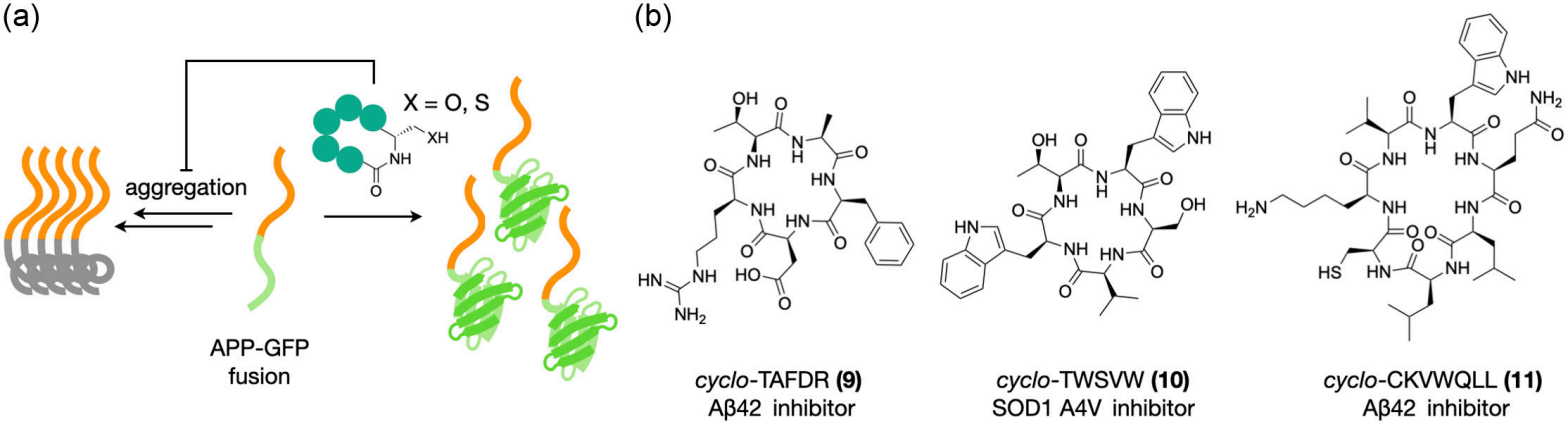
APP‐GFP reporter for selecting protein aggregation inhibitors. (a) Overview of the APP‐GFP reporter. (b) Representative cyclic peptide protein aggregation inhibitors isolated from selections using the APP‐GFP reporter.

Some APPs do not exert a toxic phenotype upon overexpression and therefore require a more generalizable biosensing strategy for identifying inhibitors of their misfolding. Genetically encoded APP‐reporter fusions can enable the direct detection of aggregation of a specific APP. In these systems, misfolding of the APP also prevents correct folding of the reporter, thereby suppressing reporter function (Figure 5a). Because they specifically read out on target protein aggregation, the use of APP‐reporter fusions also helps avoid false positives that can arise from less direct phenotypic selection strategies (such as the yeast synucleinopathy model). Matis et al. screened SICLOPPS libraries comprised of 4‐, 5‐, and 6‐mer CPs (~10^8^ transformants) for aggregation inhibitors of Aβ42 and mutant superoxide dismutase (SOD1 A4V) using APP‐GFP fusions in *E*. *coli* through fluorescence‐activated cell sorting (FACS) [21]. Although a combination of differently sized CPs containing a mixture of C, S, or T at the +1 C‐terminal extein position were interrogated, these selections predominantly enriched for 5‐mers bearing a Thr at the +1 C‐terminal extein position.

The selection against Aβ42 heavily enriched T(T/A/V)Ψ(A/D/W)R (Ψ = any canonical amino acid excluding aspartic acid and glutamic acid) as the dominant sequence motif. Two sequences, *cyclo‐*TAFDR **(9)** and *cyclo‐*SASPT, were found to inhibit Aβ42 aggregation in vitro and mitigate Aβ‐dependent neurotoxicity in primary mouse hippocampal neurons. Additionally, both CPs mitigated paralysis and Aβ deposition in *C. elegans*, with *cyclo*‐TAFDR exhibiting more pronounced effects. When the authors reapplied the same SICLOPPS library to select for inhibitors of SOD1 A4V aggregation, they observed convergence to a different sequence motif, T(Φ_1_,S)SΦ_2_W (Φ_1_ = hydrophobic amino acid, preferably A/W/F, Φ_2_ = hydrophobic amino acid, preferably V/W/F). The sequence *cyclo‐*TWSVW **(10)** was selected based on its apparent enrichment for downstream testing and was found to delay SOD1 A4V aggregation in vitro, leading to the formation of smaller aggregates.

Delivoria et al. later used the same APP‐GFP fusion approach to comprehensively screen a library of SICLOPPS heptapeptides ((C/T/S)X_6_, X = any canonical amino acid; 1.2 × 10^9^ transformants) for inhibitors of Aβ42 aggregation in *E. coli* by FACS [20]. Deep sequencing and clustering analyses of more than 4 × 10^5^ clones from the postsort population identified 416 unique CP sequences that segregated into 20 distinct clusters. Interestingly, unlike the 5‐mer CP library employed in the previous study, which favored T at the +1 C‐terminal extein position, this 7‐mer library exclusively enriched C at the same position. Two representative sequences within the most dominant clusters, *cyclo‐*CKVWQLL **(11)** and *cyclo‐*CRIVPSL, were chemically synthesized for further validation. Both CPs potently inhibited Aβ42 aggregation in ThT fluorescence assays and decreased Aβ42 aggregate accumulation and toxicity in *C. elegans*. Interestingly, *cyclo‐*CKVWQLL and *cyclo‐*CRIVPSL share no sequence similarities with the previously identified Aβ42 inhibitor *cyclo‐*TAFDR (vide supra); furthermore, none of these three CPs contain fragments of the Aβ42 sequence, which are often seen in rationally designed Aβ42 peptide inhibitors [49].

**FIGURE 6:**
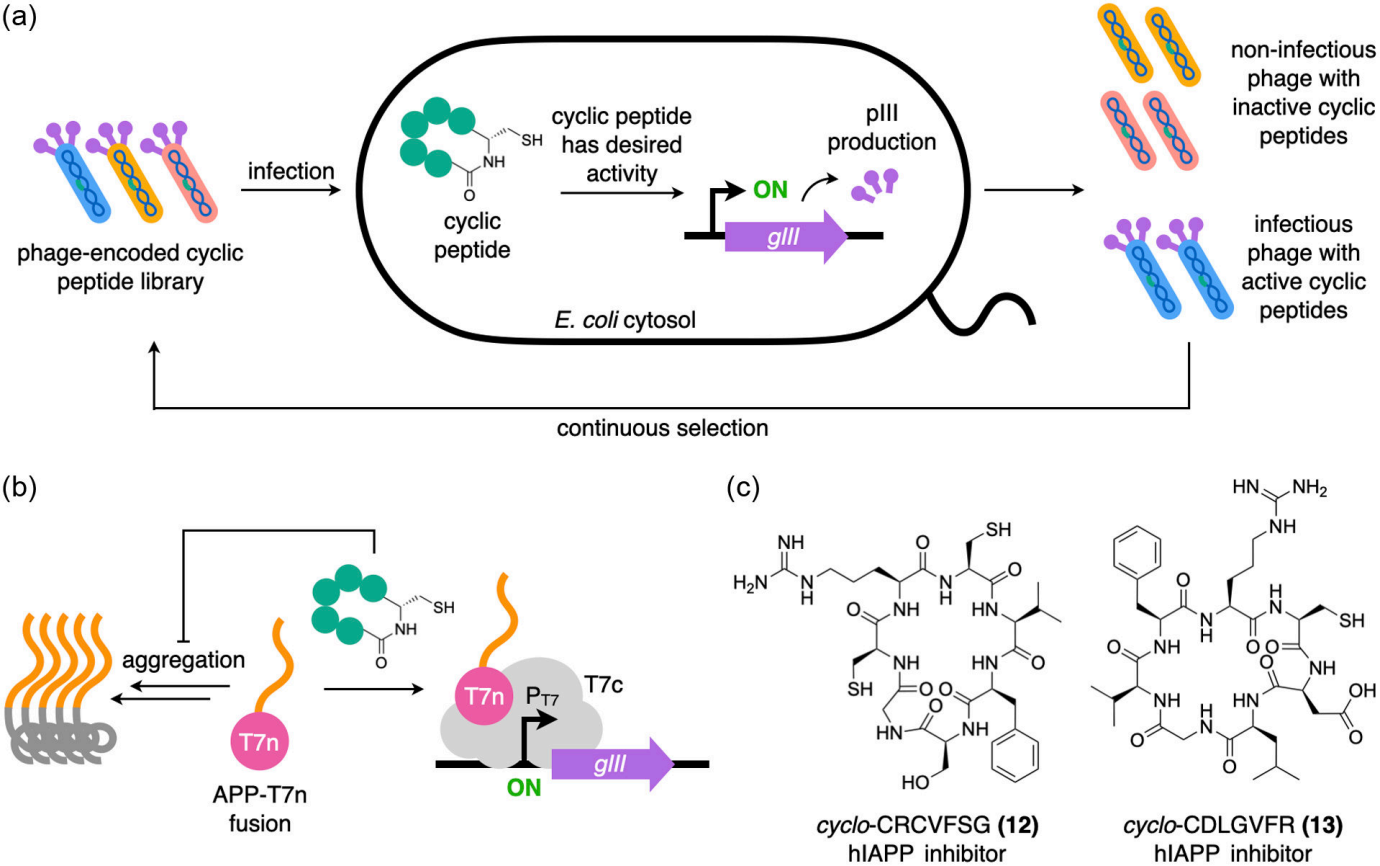
Phage‐assisted continuous selection for identifying cyclic peptide protein aggregation inhibitors. (a) Overview of the phage‐assisted selection strategy for cyclic peptide discovery. (b) Overview of an APP‐split T7 RNAP reporter for detecting protein aggregation inhibitors. (c) Cyclic peptide inhibitors for hIAPP aggregation isolated from phage‐assisted selection using the APP‐split T7 RNAP reporter.

The APP‐GFP reporter used by the studies described above illustrates the power of combining FACS with NGS for de novo CP discovery. However, this approach requires access to specialized equipment that may not be available to all research groups. Additionally, the throughput of FACS places limits on the number of CP library members that can be examined; for example, analyzing 10^9^ cells may require over a day of continuous sorting time. Phage‐assisted noncontinuous selection (PANCS) has been shown to enable the cellular selection of up to 10^11^‐member libraries in a few days using only standard molecular biology techniques and equipment [50]. We recently demonstrated that PANCS can be used for de novo CP discovery [22]. This approach employs the same M13 phage commonly used for phage display, but instead of relying on in vitro panning against an immobilized target, CPs are selected for activity in infected host cells using *gIII*, which encodes for the minor coat protein pIII, as the selection marker. By encoding the CP on the M13 genome in place of *gIII* and providing *gIII* on a host cell‐encoded plasmid under control of a genetic selection for CP activity, phage‐encoding active library members can be selected through their ability to reproduce in *E. coli* (Figure 6a).

We developed a strategy to select CP protein aggregation inhibitors using an APP‐RNAP reporter fusion. By fusing the target APP to the N‐terminal half of split T7 RNA polymerase, we linked T7 RNAP‐dependent expression of *gIII* to the inhibition of APP aggregation (Figure 6b). We used this strategy to select a phage‐encoded SICLOPPS library for inhibitors of Aβ42 and human islet amyloid polypeptide (hIAPP) aggregation. CPs enriched from selection on Aβ42 (CX_6_ library, ~3 × 10^6^ transformants) exhibited sequence motifs similar to ones observed by Delivoria et al. Interestingly, when we applied our selection to identify inhibitors of hIAPP (CX_6_ library, X = NDT codon‐encoded amino acids; ~3 × 10^6^ transformants), we observed a highly enriched sequence, *cyclo‐*CHVVGVI, that was active on both hIAPP and Aβ42. These APPs share sequence similarities in their amyloidogenic regions [51], and it is likely that *cyclo‐*CHVVGVI inhibits both through interaction with these regions. However, although hIAPP and Aβ42 are both of therapeutic interest, promiscuous inhibitors are undesirable as they can be sequestered by off‐target binding, decreasing their potency and potentially causing side effects. To improve CP selectivity, we integrated a negative selection step to remove CPs with activity on Aβ42 and isolated new sequences *cyclo‐*CRCVFSG **(12)** and *cyclo‐*CDLGVFR **(13)**, which exhibited greatly improved selectivity for hIAPP and inhibited hIAPP aggregation in vitro. Native mass spectrometry (MS) indicated that selected CPs bound hIAPP with a 1:1 stoichiometry, suggesting that they act through binding monomeric hIAPP. Notably, this work enables rapid CP enrichment through simple serial passaging of bacterial cell cultures and may represent a more accessible option for performing cellular CP selections.

**FIGURE 7:**
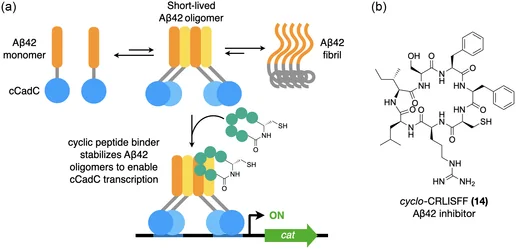
cCadC‐Aβ42 reporter for selecting AβO binders. (a) Overview of the cCadC‐Aβ42 reporter as a strategy for detecting binders to AβOs in cells. (b) Cyclic peptide inhibitor against Aβ42 aggregation isolated from a cCadC‐Aβ42 selection.

Soluble oligomers generated during the Aβ aggregation process have been implicated as one of the neurotoxic species in Alzheimer’s disease, making them a target for both therapeutic development and fundamental studies [52]. Due to their instability and heterogeneity, Aβ oligomers (AβOs) represent challenging targets. Previously, we showed that an oligomerization‐dependent transcription activator, cCadC, can be fused to Aβ to select binders to AβOs in *E. coli* [53]. Using this reporter, we selected a SICLOPPS library of heptapeptides (CX_6_, ~10^7^ transformants) for sequences that bind AβOs (Figure 7a) [54]. In this library, we employed the CfaGEP split intein (Cfa) instead of the more widely used Ssp due to Cfa’s reported improved splicing kinetics and sequence tolerance [55, 56]. After the selection and secondary screening, two sequences, CRLISFF and CFVQLFF, were identified. However, when chemically synthesized and tested for its effect on Aβ42 aggregation kinetics in vitro, *cyclo‐*CRLISFF **(14)** was found to suppress Aβ42 aggregation by inhibiting primary nucleation instead of secondary nucleation, inconsistent with an oligomer‐targeting mechanism. Although we had initially verified the splicing dependence of CRLISFF activity using a panel of previously reported intein‐inactivating mutants, the discrepancy we observed between our cellular and in vitro assays suggested that an overlooked SICLOPPS splicing intermediate might be active in our selection. From a wider panel of intein mutants, we identified an extein C1A mutant of CRLISFF, corresponding to a thioester SICLOPPS intermediate, that showed comparable activity to the original sequence and was furthermore found to selectively inhibit the secondary nucleation step of Aβ42 aggregation when purified and tested in vitro. Thus, this study highlighted both the potential and caveats (particularly in the identification of the active species) of cellular selection using SICLOPPS libraries to discover epitopes specific for soluble oligomers of APPs.

#### 2.2.4 Selecting for Protease Inhibitors

Proteases represent a large class of therapeutic targets. While most protease inhibitors function by directly blocking the active site, combination therapy involving allosteric inhibitors that target distinct regions is a promising alternative to combat rapid emergence of drug resistance. Phenotypic selections agnostic to the site of protease binding can provide a unique opportunity to identify inhibitors acting outside of the active site. A general strategy for selecting for inhibitors of a specific protease is to insert the protease’s recognition motif into a reporter protein, such as an antibiotic resistance marker or fluorescence protein. Protease inhibition thus prevents cleavage of the reporter protein, leading to selectable phenotypes such as elevated cell viability or increased fluorescence.

**FIGURE 8:**
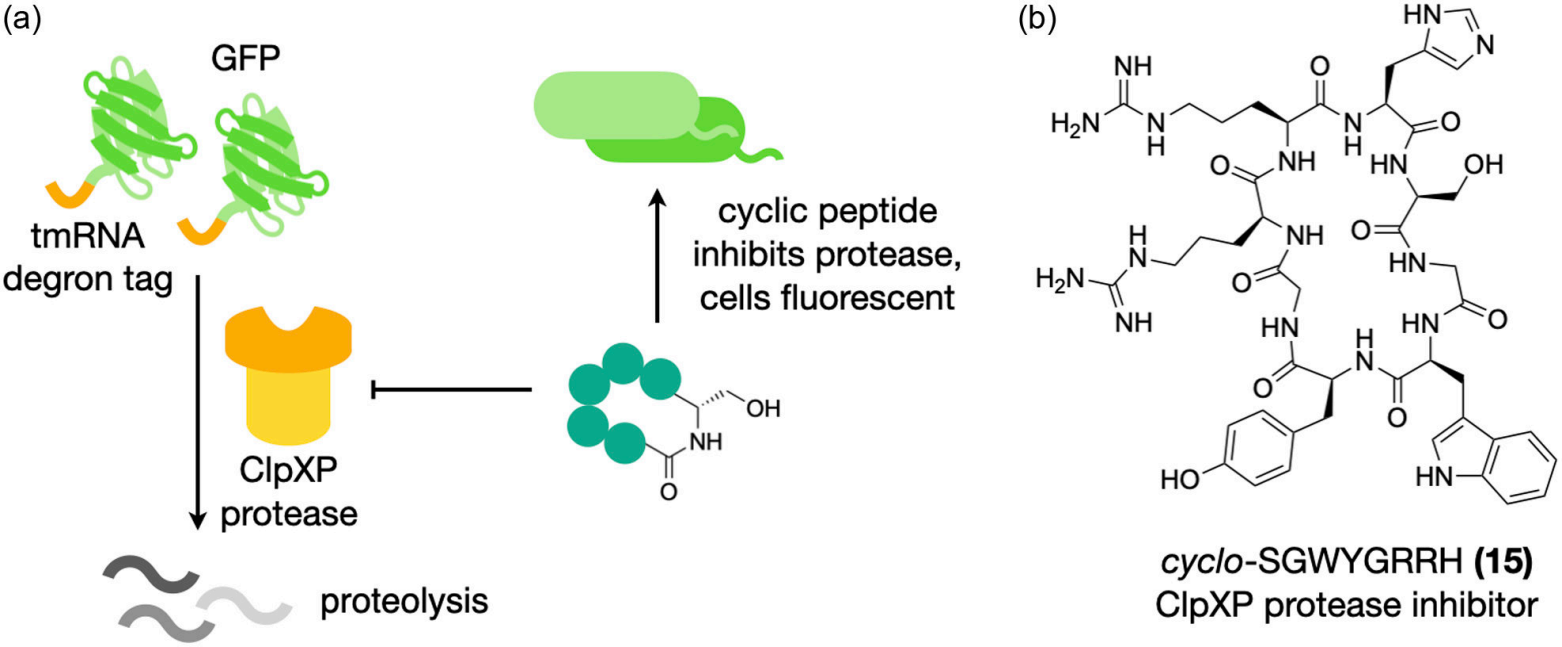
Selection for protease inhibitors using GFP as the reporter. (a) Overview of a degron tag labeled GFP reporter for detecting ClpXP protease inhibitors. (b) Cyclic peptide inhibitor for ClpXP protease isolated from a selection using the degron‐tag GFP reporter.

The bacterial protease ClpXP is responsible for degrading proteins targeted by the tmRNA tagging and degradation pathway, a process important for the virulence of several pathogenic bacteria [57]. Cheng et al. constructed a reporter to identify inhibitors of the ClpXP by fusing GFP with a C‐terminal tmRNA degron tag (Figure 8a) [58]. This reporter was used in *E. coli* to isolate ClpXP inhibitors from SICLOPPS libraries (SGWX_5_ and SGX_5_PL, X = any amino acid; ~10^6^ transformants for each library) by sorting cells with CPs that restored GFP fluorescence using FACS. Cells were sampled from the postsort bacterial populations and individually examined by fluorescence microscopy. Three clones encoding *cyclo‐*SGWYGRRH **(15)** (Figure 8b), *cyclo‐*SGSKGVLPL, and *cyclo‐*SGWRVQGPL produced similar levels of fluorescence and filamentous morphology as Δ*clpX* cells carrying the GFP‐degron reporter. Chemically synthesized *cyclo‐*SGWYGRRH was found to inhibit in vitro proteolysis of the degron‐tagged GFP by ClpXP with a K_i_ of ~136 µM. Inhibition occurred through a decrease in both ClpXP’s *K*_M_ and *k*_cat_, suggesting that the CP acts through a rare uncompetitive inhibition mechanism. This putative mechanism entails inhibition of the enzyme:substrate complex instead of the free enzyme, which was confirmed through experiments showing that *cyclo‐*SGWYGRRH suppressed ClpXP proteolysis without affecting ClpX ATPase or ClpP peptidase activity. When tested with another substrate possessing a different ClpXP recognition sequence (λ phage O protein), *cyclo‐*SGWYGRRH also increased substrate half‐life by twofold in the presence of ClpXP, suggesting that it may act as a general ClpXP inhibitor. However, *cyclo‐*SGWYGRRH showed only modest antibacterial activity when added exogenously to cultures of the Gram‐negative bacteria *Caulobacter crescentus*, with a minimum inhibitory concentration of ~220 µM, likely due to the CP’s low permeability and potency. The impermissibility of bacterial membranes presents a significant general challenge to developing antimicrobial CPs that act on intracellular targets.

**FIGURE 9:**
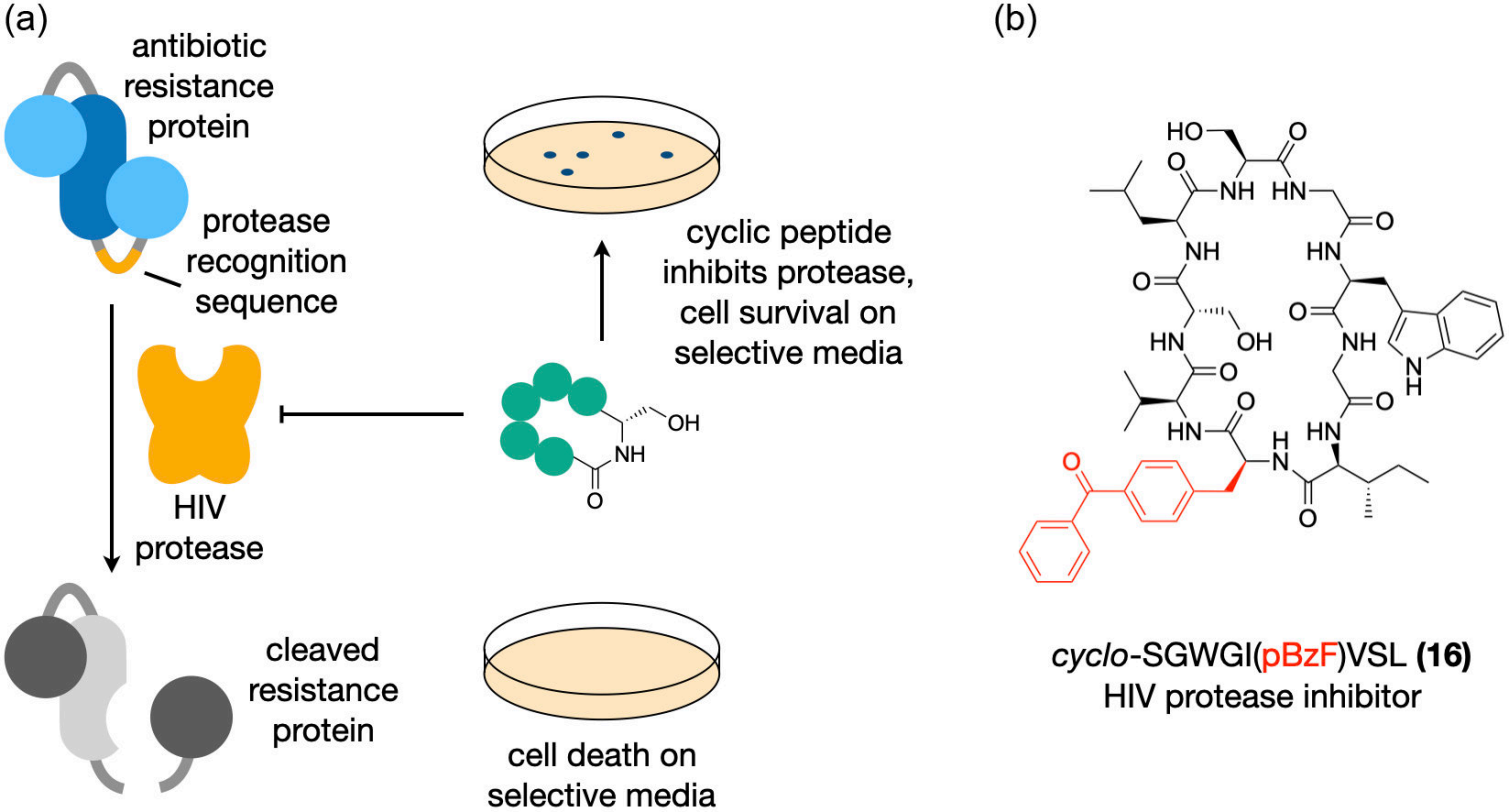
Selection for protease inhibitors using antibiotic resistance as the reporter. (a) Overview of an antibiotic resistance reporter detecting inhibition of HIV protease. (b) Cyclic peptide inhibitor for HIV protease isolated from a selection using the reporter. ncAA incorporated via genetic code expansion is labeled in red.

In another example, Young et al. incorporated the ncAA *p*‐‍benzoylphenylalanine (*p*BzF), which contains an electrophilic ketone moiety, into SICLOPPS libraries, that they then used to select HIV protease inhibitors [8]. The authors linked protease inhibition to cell survival by using a tetracycline antiporter protein with an embedded HIV protease cleavage site (Figure 9a). Cells transformed with this reporter, an engineered *p*BzF aaRS/tRNA pair, and the SICLOPPS library (SGWX_6_, X = any canonical amino acid or *p*BzF, ~3.5 × 10^8^ transformants) were plated on selective media for two rounds of selection. Notably, the TAG (amber) stop codon encoding *p*BzF was not fixed in the library but instead randomly sampled by NNK degenerate codons. Even though most of the CPs in this library would be predicted to contain only canonical amino acids, *p*BzF was highly enriched in selected sequences: out of 12 surviving colonies, six encoded *cyclo‐*SGWGI(*p*BzF)VSL **(16)** (Figure 9b), one encoded *cyclo*‐SGWVVIP(*p*BzF)I, and two encoded *cyclo‐*SGWILLGYN. SICLOPPS production of *p*BzF‐containing CPs in cells was also confirmed through SDS‐PAGE and LC/MS analysis, albeit low SICLOPPS splicing efficiencies were observed.

All three selected CPs showed inhibition of HIV protease in vitro with IC_50_s in the low µM range (~0.8–1.1 µM) and ~2 to 7‐fold higher potencies compared with their linear counterparts. The *p*BzF‐containing CPs also formed 1:1 covalent adducts with HIV protease that could be detected by LC/MS after reduction with NaBH_3_CN, suggestive of a Schiff base adduct between the *p*BzF ketone and an amine from the protease. Subsequent analysis of trypsin‐digested *cyclo‐*SGWGI(*p*BzF)VSL‐protease adducts identified K14 as the sole residue modified by this CP, and the authors found that *cyclo‐*SGWGI(*p*BzF)VSL neither modified nor inhibited a HIV protease K14R mutant. The localization of K14 outside the active site and the dimerization interface of HIV protease indicates that *cyclo*‐SGWGI(*p*BzF)VSL possesses a novel mode of inhibition. Indeed, experiments with the environmentally sensitive fluorophore 8‐anilinonapthelene‐1‐sulfonic acid (ANS) suggested that *cyclo‐*SGWGI(*p*BzF)VSL may act through promoting HIV protease unfolding or aggregation.

#### 2.2.5 Selecting Antibacterials With New Mechanisms of Action

Although more than 250 antibiotics are currently available for clinical use, most function against a limited set of targets with increasingly reported drug resistance, underscoring the urgent need for antibacterials with novel mechanisms of action (MoAs). Whole‐cell screening of small‐molecule libraries is historically successful but suffers from a high rediscovery rate of compounds with the same MoAs as existing antibiotics. In contrast, a fully bottom‐up genetic approach, where inhibition of the putative target is sensed by a customized selection system, would enable high‐throughput MoA‐guided antibiotic discovery when applied to cellular selection. A few studies have explored this direction in conjunction with SICLOPPS to select for antibacterial CPs [58, 59, 60, 61].

**FIGURE 10:**
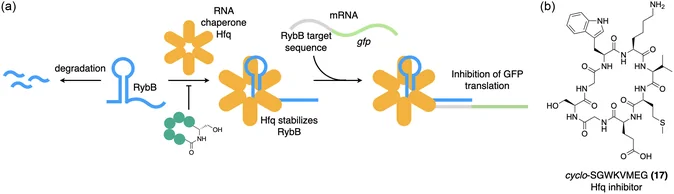
Selection for inhibitors against bacterial RNA chaperone Hfq. (a) Overview of the selection strategy. (b) Cyclic peptide inhibitor for Hfq isolated from the selection.

El‐Mowafi et al. selected inhibitors against the bacterial RNA chaperone Hfq, whose activity has been shown to be associated with virulence [60]. Hfq facilitates the silencing of target mRNAs by the sRNA RybB, which is otherwise quickly degraded in the absence of the Hfq chaperone [62]. By embedding the RybB target sequence within the 5' region of an mRNA encoding a fluorescence protein, the authors translated Hfq inhibition into an increase in fluorescence signal (Figure 10a). A SICLOPPS library (SGWX_5_, X = any canonical amino acid; ~2 × 10^6^ transformants) was applied to this system for selection of Hfq inhibitors using FACS, identifying the sequence *cyclo‐*SGWKVMEG **(17)** (Figure 10b). When expressed in cells, this construct showed ~3‐fold inhibition of Hfq and RybB‐mediated degradation of target mRNA as measured by Northern blot and sensitized *E. coli* to the antibiotics benzalkonium chloride (twofold decrease in MIC) and novobiocin (fourfold decrease in MIC). When chemically synthesized and tested in a gel mobility shift assay, *cyclo*‐SGWKVMEG inhibited the Hfq:RybB interaction with a *K*_i_ of ~110 µM; in comparison, the *K*_d_ of the Hfq:RybB interaction has been estimated to be ~90 nM. *cyclo*‐SGWKVMEG increased the fluorescence of *E. coli* transformed with the Hfq fluorescent reporter when added exogenously to bacterial cultures at high concentrations (500 µM) but failed to exhibit antibacterial activity.

El‐Mowafi et al. also targeted the alternative sigma factor *σ* ^E^ which has been shown to induce virulence in response to cell envelope stress in various pathogens [61, 63]. The authors modified the reporter system they employed to identify Hfq inhibitors (Figure 10a) by placing RybB expression under the control of *σ* ^E^. Inhibition of *σ* ^E^ then leads to decreased RybB‐mediated silencing of the target fluorescent protein‐encoding mRNA and increased cellular fluorescence. Using the same SICLOPPS library and selection workflow used in their Hfq inhibitor selection (vide supra), the authors identified a highly active library member, which surprisingly encoded the sequence *cyclo*‐SGWLGS*P (* = TAG stop codon). LC–MS/MS analysis conducted on cell lysates expressing the SICLOPPS construct revealed production of *cyclo‐*SGWLGSKP in cells, indicating that lysine was incorporated at the stop codon. Expression of *cyclo‐*SGWLGSKP not only increased fluorescence of *E. coli* encoding the reporter but also reduced viability and caused extended filamentation. Chemically synthesized *cyclo*‐SGWLGSKP bound *σ* ^E^ with a *K*_d_ of 32 µM (measured by fluorescence anisotropy) and inhibited σ^E^‐mediated transcription in vitro (IC_50_ of 270 µM). However, as with the Hfq inhibitor *cyclo‐*SGWKVMEG, *cyclo‐*SGWLGSKP also failed to display antibacterial activity when exogenously added to bacterial cultures.

In summary, these studies demonstrate how cellular selection for MoA‐guided discovery of antibacterial CPs can lead to hits active on nontraditional targets but also highlight the outstanding challenge of translating preliminary hits into useful lead compounds with sufficient potency and permeability, especially for Gram‐negative bacteria, which feature a lipopolysaccharide barrier and a less permeable outer membrane.

## 3 Sidechain‐to‐Sidechain Crosslinked Cyclic Peptides

**FIGURE 11:**
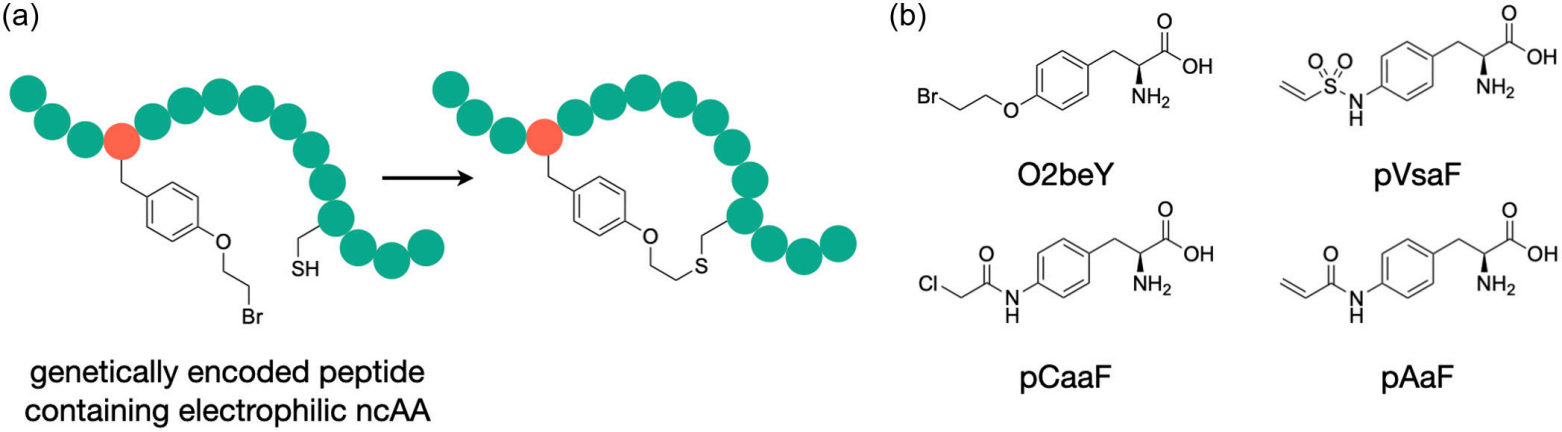
Macrocyclic organo‐peptide hybrids (MOrPHs). (a) Production of sidechain‐to‐sidechain crosslinked cyclic peptides in cells via reaction between an electrophilic ncAA and cysteine residue. (b) Representative electrophilic ncAAs used in MOrPH for intracellular production of genetically encodable cyclic peptides.

In addition to head‐to‐tail cyclization enabled by the SICLOPPS method, side‐chain‐to‐side‐chain cyclization can also be used to produce genetically encoded CP libraries in cells. Macrocyclic organo‐peptide hybrids (MOrPHs), for example, utilize reactions between a nucleophilic amino acid (usually cysteine) and an electrophilic ncAA to produce sidechain‐to‐sidechain cyclized peptides (Figure 11a) [64]. In MOrPHs, the target peptide sequence is genetically encoded, and the electrophilic ncAA is incorporated by its corresponding aaRS/tRNA pair. Several ncAAs have been demonstrated to enable production of CPs of varying ring sizes (up to 11 amino acids) in *E. coli* cells (Figure 11b) [65]. Although MOrPH libraries have been successfully implemented for binder discovery using phage display [66], their application to cellular selections has yet to be reported.

**FIGURE 12:**
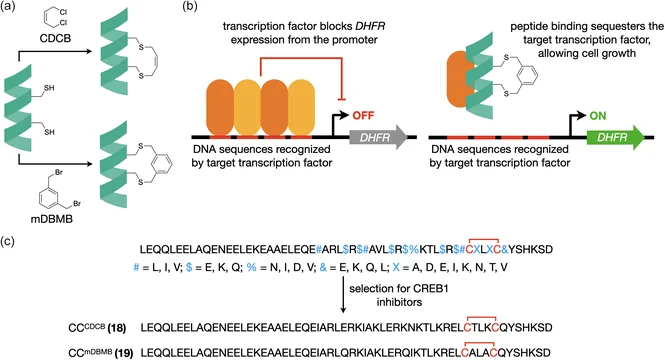
Stapled α‐helical peptides and cellular selection for transcription factor inhibitors. (a) Production of sidechain‐to‐sidechain crosslinked α‐helical peptides in cells via exogenously supplemented bis‐alkylating electrophiles. (b) Overview of the transcription block survival strategy for selecting inhibitors against transcription factors. (c) Library design and enriched cyclic peptide inhibitors for CREB1.

Recently, Brennan et al. reported the use of bis‐alkylating electrophiles to crosslink proximal cysteines for CP production in *E. coli* with a focus on identifying stapled α‐helical peptide inhibitors of transcription factors (Figure 12) [10]. Directly supplementing media with *cis*‐1,4‐dichloro‐2‐butene (CDCB) or 1,3‐dibromomethylbenzene (mDBMB) enabled intracellular cyclization of peptides by cross‐linking two cysteines separated by up to six residues (Figure 12a). To detect sequences capable of inhibiting transcription factor activity, the authors employed a “transcription block survival” selection where the expression of dihydrofolate reductase (DHFR) is blocked by transcription factor binding to consensus DNA sequences arrayed at the promoter (Figure 12b) [67]. Peptide libraries (~6 × 10^7^ transformants) based on a 59‐mer sequence previously designed to interact with the oncogenic transcription factor CREB1 were generated with a conserved motif CXLXC (X = A/D/E/I/K/N/T/V) for cross‐linking (Figure 12c) [68]. Several rounds of passaging cells encoding this library and the transcription block survival selection in media supplemented with either CDCB or mDBMB enriched two peptides, CC^CDCB^ **(18)** and CC^mDBMB^ **(19)**, that show high sequence homology to each other. Both CC^CDCB^ (K_d_ ~ 41 nM) and CC^mDBMB^ (K_d_ ~ 53 nM) bound CREB1 more tightly than their linear counterparts (K_d_ ~ 125 nM and 140 nM, respectively) in ITC and displayed strong inhibitory activity (IC_50_ ~ 247 nM and 511 nM, respectively) against the CREB1:CRE DNA interaction in fluorescence polarization assays. To enable delivery into mammalian cells for testing, CC^CDCB^ was fused to a cell‐penetrating peptide. The resulting fusion reduced cell viability (IC_50_ ~ 15 µM), inhibited CREB1 transcriptional activation (IC_50_ ~ 7 µM), and decreased the transcription level of CREB1‐regulated genes in A375 human melanoma cells, suggesting promising antagonism of CREB1. This study provides a new sidechain‐to‐sidechain cyclization method that can be applied to produce stapled α‐helical peptides as genetically encodable scaffold for cellular selection of CPs. Considering the therapeutic significance of inhibitors for oncogenic transcription factors [69], the selection system used in this study may hold great promise for future investigation.

## 4 Ribosomally Synthesized and Posttranslationally Modified Peptides (RiPPs)

**FIGURE 13:**
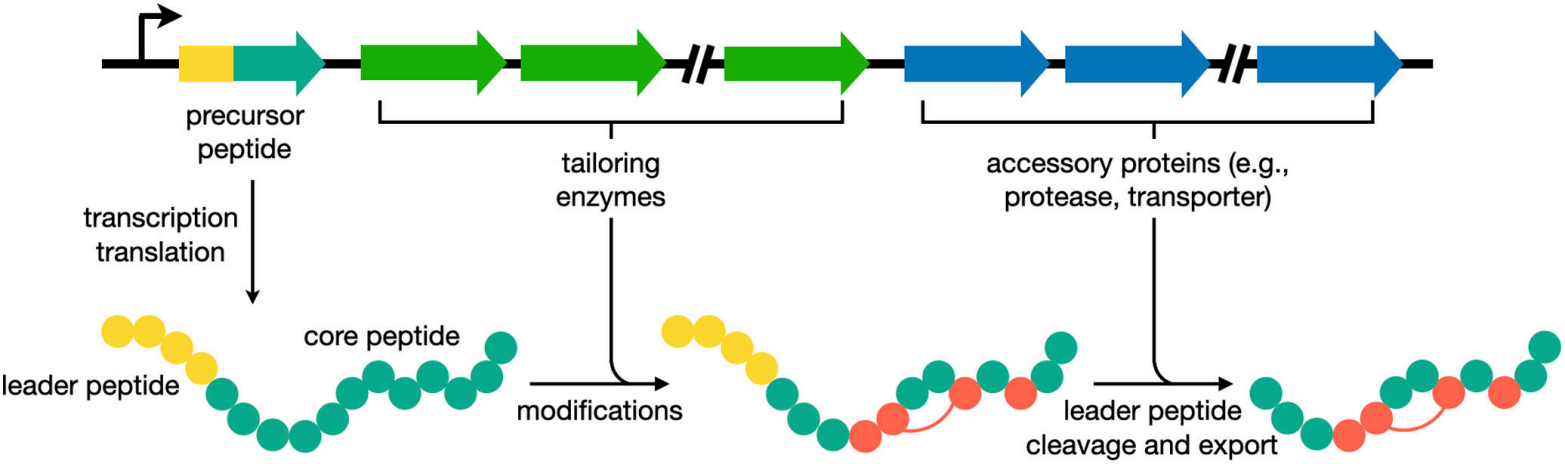
Biosynthesis of RiPPs.

RiPPs are a diverse class of natural products found across all domains of life. RiPPs can possess valuable antimicrobial, anticancer, and immunosuppressant activities, making them a promising source of compounds for therapeutic discovery [70, 71]. RiPPs are commonly divided into families according to the types of posttranslational modifications their core sequences undergo. More than 40 such subclasses have been described, and this number continues to grow rapidly. Despite this diversity, all RiPPs undergo a conserved sequence of processing events for maturation (Figure 13). RiPPs are genomically encoded as a precursor peptide, which frequently consists of an N‐terminal leader peptide, a core peptide, and, in some cases, a C‐terminal follower peptide. Once translated by the ribosome, the precursor peptide is modified by tailoring enzymes that install unique posttranslational modifications whose nature define each family of RiPPs. Finally, the leader peptide is proteolytically cleaved from the precursor peptide, yielding the mature RiPP. (For a more comprehensive review on RiPPs, please see references [72, 73, 74].) Their genetic encodability and biosynthetic modularity render RiPPs attractive for the generation of combinatorial libraries that can be employed for de novo discovery. We highlight representative examples where RiPP libraries have been successfully adapted for cellular selection of bioactive CPs.

### 4.1 Lanthipeptides

**FIGURE 14:**
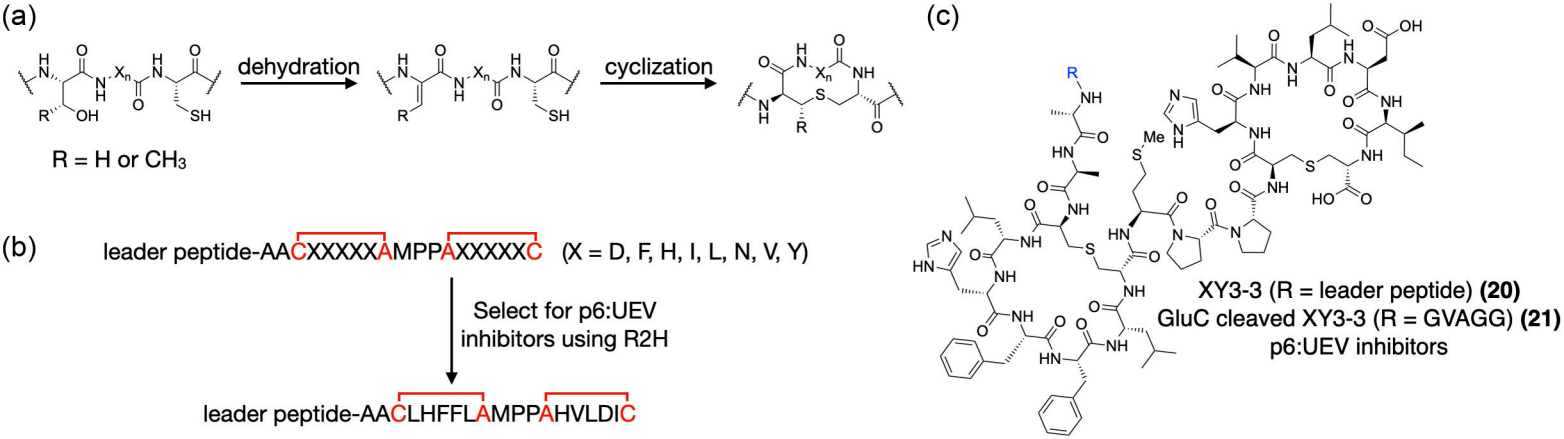
Cellular selection of genetically encoded lanthipeptide libraries. (a) Production of lanthipeptides via dehydration and cyclization of the precursor peptide. (b) Library design and enriched sequence of a lanthipeptide inhibitor for p6:UEV interaction. (c) Structure of the lanthipeptide inhibitors for p6:UEV interaction isolated from a R2H selection.

Lanthipeptides are characterized by the presence of sulfur‐to‐carbon thioether crosslinks, termed lanthionines (Lan) and methyllanthionines (MeLan), that create sidechain‐to‐sidechain macrocycles [75]. During lanthipeptide maturation, a dehydratase first converts serine or threonine residues in the core peptide into dehydroalanine or dehydrobutyrine, respectively. Subsequently, a cyclase catalyzes the stereoselective 1,4‐conjugate addition of cysteine into these dehydro‐amino acids to generate the Lan and MeLan adducts (Figure 14a). Finally, the leader sequence is cleaved, and the mature lanthipeptide is exported.

Among characterized lanthipeptide biosynthetic enzymes, ProcM, which mediates prochlorosin biosynthesis, is notable for its ability to function as a single tailoring enzyme catalyzing both the dehydration and cyclization reactions [76]. Moreover, ProcM exhibits remarkable sequence tolerance toward its core peptide substrates and is readily expressed in *E. coli*, making it well‐suited for generating large genetically encoded libraries for cellular selection. In a pioneering study, Yang et al. generated and selected lanthipeptide libraries based on the core peptide found in the prochlorosin ProcA2.8 in *E. coli* (Figure 14b) [11]. These libraries featured a randomized core sequence, AACX_5_SMPPSX_5_C (X = amino acids encoded by NWY codons, chosen due to the exclusion of cysteine, serine, and threonine) and were coexpressed with ProcM but without a leader peptide protease or transporter, ensuring that biosynthesized lanthipeptides remained in the *E. coli* cytosol. MS analysis of peptides produced by 33 randomly selected clones from a library of ~10^6^ unique sequences confirmed that all 33 contained the expected Lan crosslinks, underscoring the robustness and sequence tolerance of ProcM.

The authors selected the lanthipeptide library for inhibitors of the p6:UEV interaction using the same reverse two‐hybrid (R2H) used to identify the inhibitor *cyclo‐*SGWIYWNV from SICLOPPS libraries (vide supra). This experiment yielded 80 surviving colonies, from which one sequence was identified to pass secondary screening and show inhibitory activity specifically for the p6:UEV interaction. This isolated peptide, **XY3‐3 (20)**, bound UEV with *K*_d_ ~ 16 µM and inhibited the p6:UEV interaction with an IC_50_ of ~3.6 µM. A leader peptide‐less version of **XY3‐3** produced by endoprotease Glu‐C cleavage **(21)** showed improved affinity for UEV (*K*_d_ ~ 4 µM) and p6:UEV inhibition (IC_50_ ~ 1.9 µM), suggesting that its activity is driven by the core peptide sequence. Interestingly, **21** did not displace a PTAP‐containing peptide from UEV in a competitive fluorescence polarization assay, indicating it binds to a region on UEV outside of the PTAP binding pocket. Finally, to test its ability to disrupt viral budding, **21** was modified with an N‐terminal cysteine and conjugated to the cell‐penetrating Tat peptide. The resulting construct inhibited virus‐like particle formation and TSG101‐mediated degradation of the epidermal growth factor receptor in 293T cells. Compared to the SICLOPPS‐derived inhibitor *cyclo‐*SGWIYWNV, both **XY3‐3** and **21** exhibited improved potency despite being identified from a similarly sized library, demonstrating the promising utility of lanthipeptides for inhibiting PPIs and highlighting the broader importance of exploring scaffolds beyond head‐to‐tail CPs in cellular selections.

### 4.2 Ranthipeptides

Radical non‐α thioether peptides (ranthipeptides) contain thioether cross‐links between cysteine and the β‐carbon or γ‐carbon of an acceptor residue [77]. These modifications are catalyzed by radical S‐adenosylmethionine (rSAM) maturases. Ranthipeptide precursors typically feature cysteine‐rich sequences with repeated motifs, such as CX_3_(D/E). Despite this conservation, rSAM maturases such as PapB tolerate variations in core peptide sequence and length, enabling the production of diverse thioether‐linked multicyclic peptides for selection [78].

**FIGURE 15:**
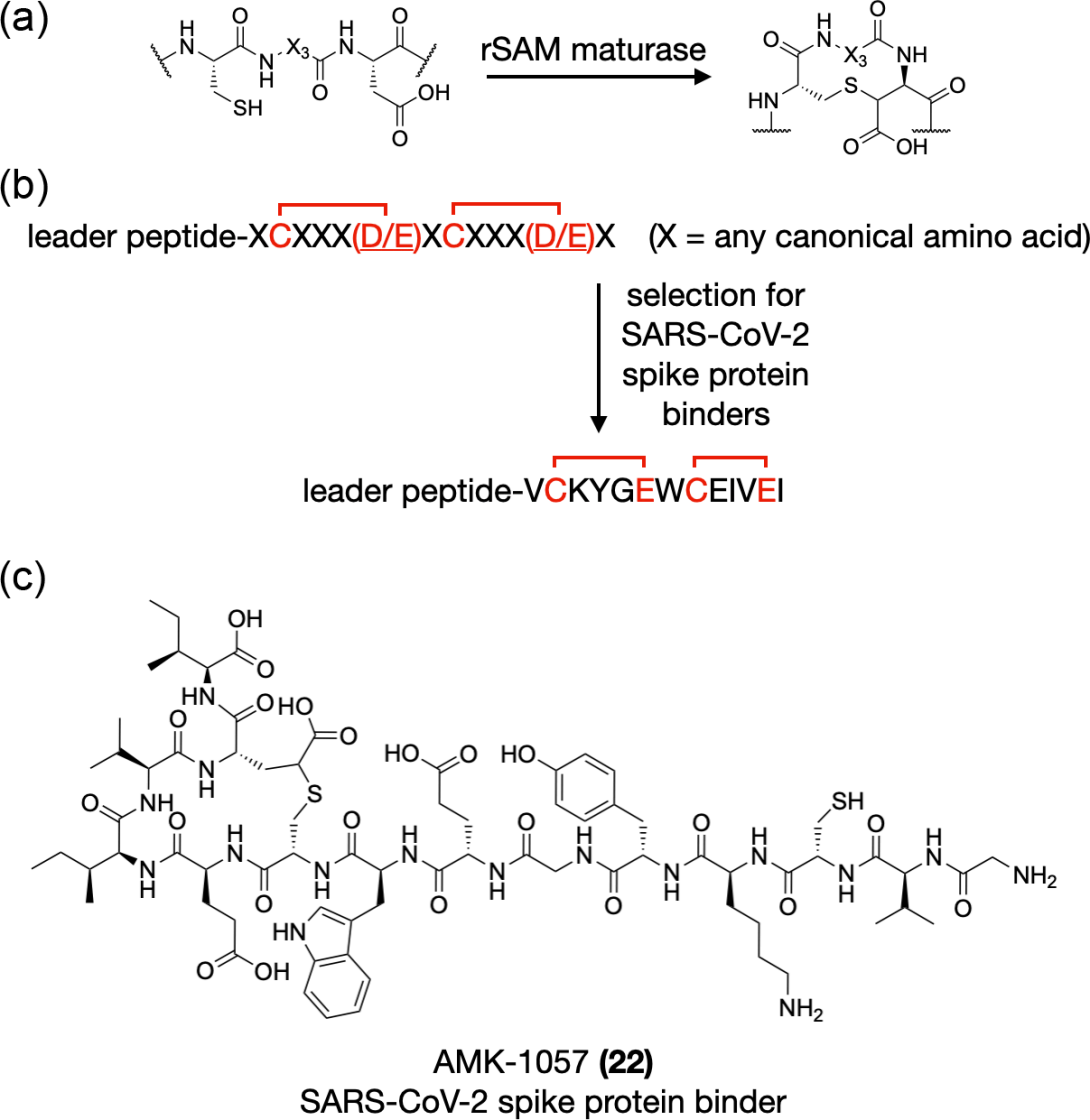
Cellular selection of genetically encoded ranthipeptide libraries. (a) Production of ranthipeptides catalyzed by rSAM maturase. (b) Library design and enriched sequence of a ranthipeptide binder for SARS‐CoV‐2 spike protein. (c) Structure of the ranthipeptide binder for SARS‐CoV‐2 spike protein.

**FIGURE 16:**
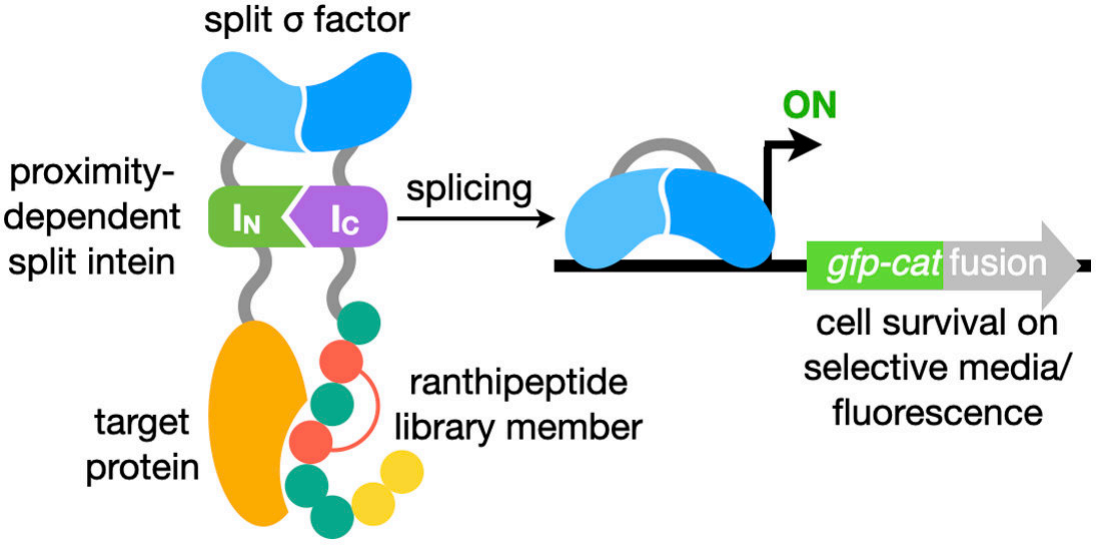
Overview of the selection strategy for protein binders via the use of split intein and split σ factor.

King et al. developed a ranthipeptide library based on the natural substrate PapA by using PapB to modify a randomized core sequence of XCX_3_(D/E)XCX_3_(D/E)X (X = any of the 20 canonical amino acids; ~10^8^ transformants) (Figure 15) [12]. To assess PapB activity, the authors performed MS analysis of 19 random clones and found that 14 produced peptides with masses consistent with one or two thioether cross‐links. This library was applied in *E. coli* to select for binders of the SARS‐CoV‐2 spike protein using a new genetic selection the authors created based on the target‐binding‐driven reconstitution of a split σ factor. In this system, the N‐terminal half of the *σ* factor (*σ* ^N^) is fused to the spike protein via a split intein (int^N^), while the ranthipeptide precursor is fused to the C‐terminal half of the *σ* factor (*σ* ^C^) via the complementary split intein (int^C^). Interaction between the ranthipeptide library member and the spike protein facilitates intein association and full‐length σ factor production through trans‐splicing, which then drives the expression of a GFP‐chloramphenicol acetyltransferase fusion selectable marker (Figure 16).

After three rounds of positive selection with increasing chloramphenicol concentrations, NGS identified 20 sequences representing >1% of the final population. Only one candidate showed specific activity toward the SARS‐CoV‐2 spike protein in secondary *E. coli* screens. This peptide (core sequence: VCKYGEWCEIVEI) was purified in the presence of PapB; however, MS and fragmentation analysis revealed a single thioether crosslink between C2 and E6, rather than the two‐crosslink scaffold the library was designed around. The corresponding ranthipeptide, AMK‐1057 **(22)**, bound the spike protein with a *K*_d_ of ~990 nM (measured by BLI). Neither the leader peptide nor an unmodified variant showed binding, confirming the importance of the modified core. Although the original intent of the study was to identify peptide inhibitors of the spike protein:ACE2 interaction, competitive BLI experiments indicated that AMK‐1057 binds a region distinct from the ACE2 binding site.

At present, the RiPPs that have been employed in cellular selections remain largely confined to a small number of well‐studied families. This imbalance reflects the limited characterization of in vivo substrate tolerance for many RiPP enzymes. Consequently, although several additional RiPP families likely possess strong potential for cellular selection, they have yet to be explored in this context [79, 80, 81].

## 5 Summary and Outlook

Although cellular selections have yielded many bioactive CPs for a wide range of targets, as discussed above, there remain limitations that, if addressed, could greatly enhance the utility of these technologies for molecular discovery.

First, more target classes are awaiting selection systems compatible with future applications in cellular selections. While any protein that can be expressed in cells is theoretically a candidate target, selecting for inhibitory activity is not yet as generalizable as selecting for target binding as the former requires robust systems capable of translating the desired activity into a selectable phenotype. Therefore, expanding the target scope will likely require the development of new selection systems compatible with different target classes. Even for existing selection systems, further engineering may be necessary to investigate additional targets. For instance, the R2H system has been almost exclusively applied to parallel PPIs, and the APP‐reporter systems do not appear to tolerate APPs with slow aggregation kinetics. Additionally, greater extension of CP cellular selections to eukaryotic (or, ideally, mammalian) cells is required to explore an increasing number of drug targets that are incompatible with *E. coli*, which again demands the development of suitable selection systems for these new hosts.

Second, although CP hits identified from cellular selections are more likely to possess biological activity, they often lack the potency required for lead compound development. Aside from the potentially suboptimal ability of the selection systems to isolate highly active variants, a major cause of low hit potency is arguably insufficient chemical diversity, which encompasses both the CP library size and the diversity of sidechains and scaffolds. At present, CP library size is constrained by DNA transformation efficiency (which typically enforces a ceiling of ~10^9^ variants). In situ mutagenesis may enable the expansion of library sizes past the transformation limit, but successful implementation of these strategies has yet to be reported. Notably, several studies have demonstrated that incorporating judiciously chosen ncAAs (e.g., ones bearing electrophilic warheads or substrate mimics) into CP libraries can yield hits with increased potency. Employing different CP scaffolds or topologies may similarly improve hit potency through the expansion of chemical diversity, as suggested by studies examining RiPP libraries in cellular selections. With recently established methods of sidechain‐to‐sidechain cyclization for producing genetically encodable CP libraries in cells, more CP scaffolds, such as stapled α‐helical peptide and potentially MOrPHs, are also becoming available for application to cellular selections.

Third, due to the phenotypic nature of cellular selection, false positives that “cheat” in the selection system can pass the preliminary selection along with true hits. Thus, it is often necessary to implement secondary screening/selection steps or integrate negative selection against off‐targets to purge false positives and validate desired biological activity. Likewise, using a selection system that directly links target inhibition to the selectable phenotype in place of an indirect phenotypic selection (e.g., employing an APP‐reporter fusion vs. the yeast synucleinopathy model) can help reduce the false positive rate. Therefore, while cellular selection can suffer from false positives like other high‐throughput CP discovery methods, researchers can often leverage established workflows and practices to mitigate this issue.

Finally, it is worth noting that membrane permeability is not being evaluated during cellular selection as production of CPs happens inside the cytosol. Additional chemical modifications, such as cell‐penetrating peptide conjugation, have been employed in several studies to increase membrane permeability of preliminary hits to enable in vitro assays in mammalian cells. Although some backbone‐modified (e.g., amide *N*‐methylated) peptides have increased membrane permeability, their efficient ribosomal production in cells remains challenging, and they cannot currently be employed in cellular selections. Therefore, although cellular selection can rapidly identify bioactive CPs, further medicinal chemistry is often necessary to improve important pharmacokinetic parameters of the hits, including membrane permeability and eventually bioavailability.

In summary, cellular selection is a powerful strategy that offers unique advantages for the de novo discovery of bioactive CPs, especially for targets difficult to obtain and handle in vitro. Moving forward, efforts focused on the development of robust selection systems for a broader range of targets and methods to generate more diverse CP libraries will help take full advantage of modularity and engineerability of this approach to enable CP discovery.

## Funding

National Institutes of Health (R35 GM159851).

## Conflicts of Interest

The authors declare no conflicts of interest.

## Supporting information

Supplementary Material
