# Supplementary material for: De Novo Discovery of Bioactive Cyclic Peptides via Cellular Selection of Genetically Encoded Libraries
Source: Chembiochem. 2026 Sep 23;27(18):e70532. doi: 10.1002/cbic.70532 (PMC13599706; doi:10.1002/cbic.70532)
Supplement: Supplementary file 1 [file CBIC-27-e70532-s001.pdf]

Reference	CP class	Host organism	CP library design	Enriched sequence [b]	Structure of optimized and *in vitro/in cellulo* validated peptide [c]	Apparent [d] and theoretical [e] library size	Target	Selection system	Potency of chemically re-synthesized peptide	Cell permeability of chemically re-synthesized peptide
[1]	Head-to-tail CP	Human B cell	SXXXX [a]	SRVEI	N/A	2.7×10^5 (apparent) and $\sim 1 \times 10^5$ (theoretical)	Interleukin-4 mediated IgE class switching	See the reference [f]	N/A	N/A
[2]	Head-to-tail CP	*E. coli*	CXXXXX	CRYYNV	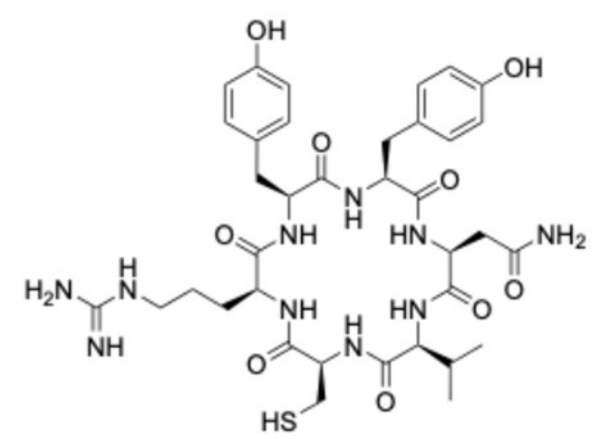	~1E8 (apparent) and ~3.3E7 (theoretical)	Ribonucleotide reductase	R2H	Bound the ribonucleotide reductase small subunit with a K_d of ~53 μM.	Not tested
[3]	Head-to-tail CP	*E. coli*	CXXXXX	CRYFNV	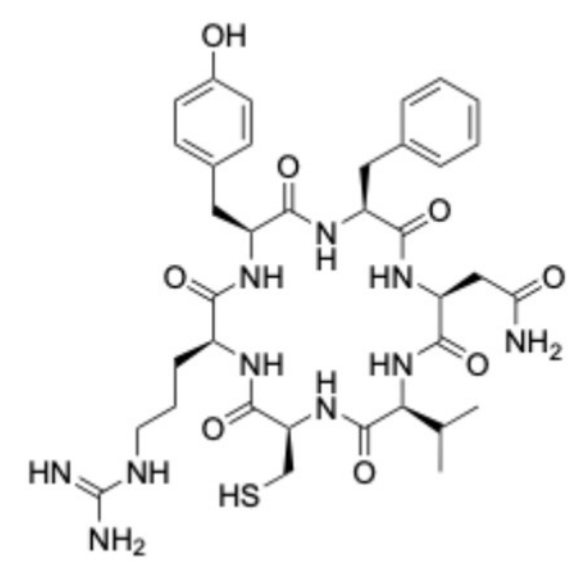	$\sim 1 \times 10^7$ (apparent) and 3.3×10^7 (theoretical)	Aminoimidazole-4-carboxamide ribonucleotide transformylase	R2H	Inhibited aminoimidazole-4-carboxamide ribonucleotide transformylase with a K_i of ~17 μM.	Not tested
[4]	Head-to-tail CP	*E. coli*	SGWXXXXX	CGWIYWNV	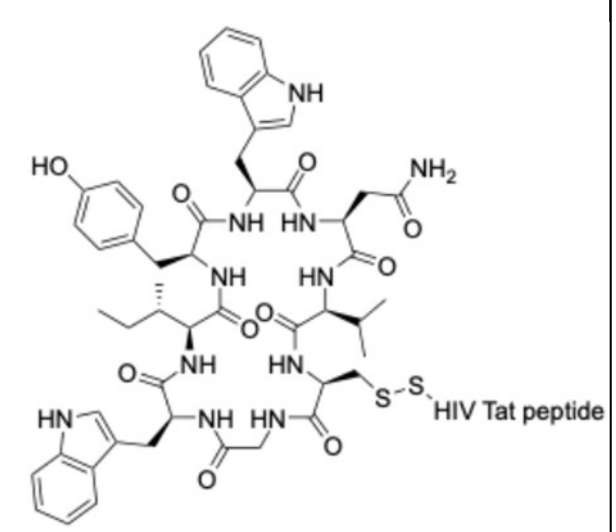	$\sim 4 \times 10^8$ (apparent) and $\sim 3.3 \times 10^7$ (theoretical)	p6 subunit of HIV Gag protein:UEV domain of human TSG101 protein (p6:UEV) interaction	R2H	Inhibited Gag-mediated viral-like particle production with an IC_{50} of ~7 μM.	Permeable to 293T cells when fused to Tat peptide
[5]	Head-to-tail CP	*E. coli*	SGWXXXXX	SGWYVRNM	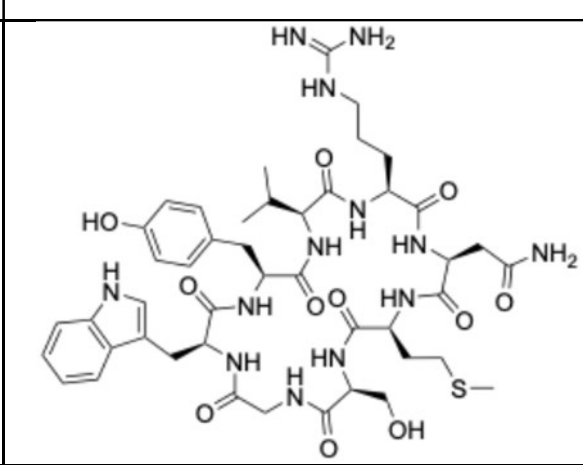	2.4×10^7 (apparent) and $\sim 3.3 \times 10^7$ (theoretical)	*E. coli* dam methyltransferas e	See the reference	Inhibited *E. coli* dam methyltransferase with an IC_{50} of ~50 μM.	Not tested

[6]	Head-to-tail CP	*E. coli*	SGWXXXXX	SGWYGRRH	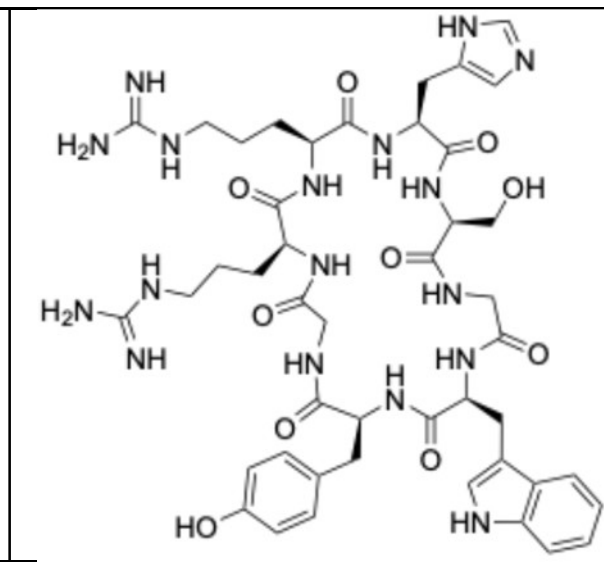	~1x10^{6} (apparent) and 3.3x10^{7} (theoretical)	Bacterial ClpXP protease	Protease-cleavable reporter	Inhibited ClpXP protease with a K_i of ~136 μM and inhibited the growth of *Caulobacter crescentus* with an MIC of ~219 μM.	Suggested to be permeable to *Caulobacter crescentus* cells to some degree
[7]	Head-to-tail CP	*S. cerevisiae*	CXXXXXXX	CPWCSTRV	N/A	5x10^{7} (apparent) and ~3x10^{10} (theoretical)	α-synuclein	Yeast synucleinopathy model	N/A	Not tested
[8]	SICLOPPS lariat peptide	*S. cerevisiae*	SXXXXXXXEY-Ic	SRSWDLPGEY-Ic	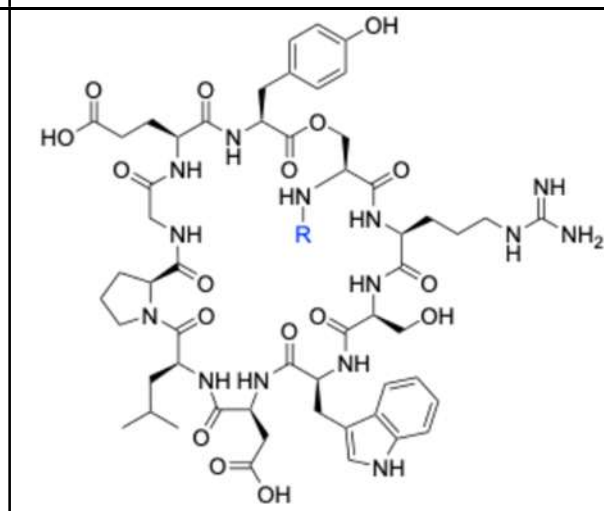 (R = I_C)	5x10^{6} (apparent) and ~3x10^{10} (theoretical)	*E. coli* LexA	Y2H	Bound LexA with a K_d of ~37 μM.	Not tested
[9]	Head-to-tail CP	*E. coli*	SGWXXXXXX (X = any canonical amino acid and *p*-benzoylphenylalanine)	SGWGI*VSL (* = *p*-benzoylphenylalanine)	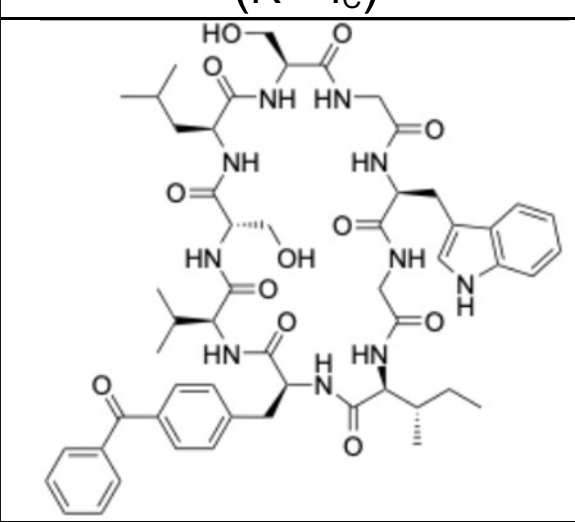	3.5x10^{8} (apparent) and ~1x10^{9} (theoretical)	HIV protease	Protease-cleavable reporter	Inhibited HIV protease with an IC_{50} of 0.96 μM.	Not tested
[10]	Head-to-tail CP	*E. coli*	SGWXXXXXX	SGWTVVRMY	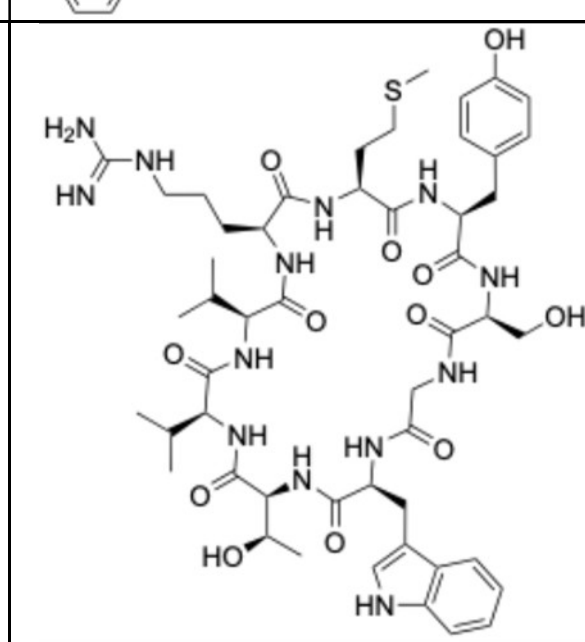	~1x10^{9} (theoretical)	C-terminal binding protein (CtBP)	R2H	Bound CtBP1 with a K_d of 3-11 μM and inhibited CtBP2 homodimerization with an IC_{50} of ~19 μM.	Permeable to mammalian cells (COS-7) when fused to Tat peptide (first serine replaced with cysteine to form disulfide bond for conjugation)

[11]	Head-to-tail CP	*E. coli*	CXXXXX	CLLFVY	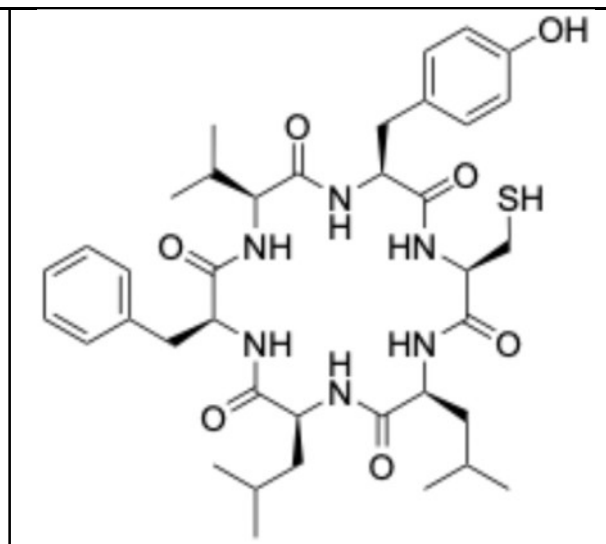	~5x10^7 (apparent) and ~3.3x10^7 (theoretical)	Hypoxia inducible factor-1α (HIF-1α):HIF-1β interaction	R2H	Bound HIF-1α with a K_d of 0.124 μM and inhibited HIF-1α:HIF-1β interaction with an IC_{50} of 1.3 μM.	Permeable to mammalian cells (U2OS and MCF-7) when fused to Tat peptide
[12]	Head-to-tail CP	*E. coli*	SGWXXXXX	SGWKVMEG	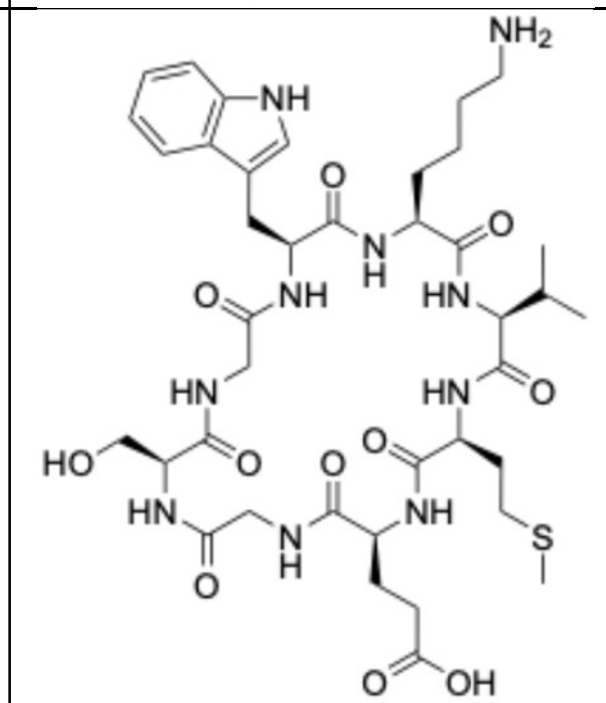	2x10^6 (apparent) and ~3.3x10^7 (theoretical)	Bacterial RNA chaperone Hfq	See the reference	N/A	Suggested to be permeable to *E. coli* cells to some degree
[13]	Head-to-tail CP	*E. coli*	SGWXXXXX	SGWLGSKP	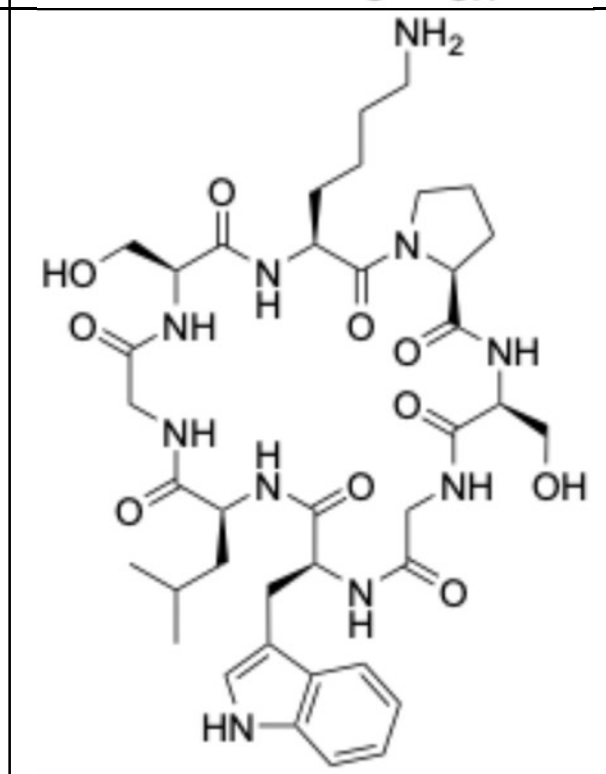	2x10^6 (apparent) and ~3.3x10^7 (theoretical)	Bacterial sigma factor σE	See the reference	Inhibited σE-dependent transcription with an IC_{50} of ~270 μM.	Impermeable to *E. coli* cells
[14]	Head-to-tail CP	*E. coli*	SIIDSAGXXXXXXGASTSESG	SIIDSAGVFLCGCGASTSESG	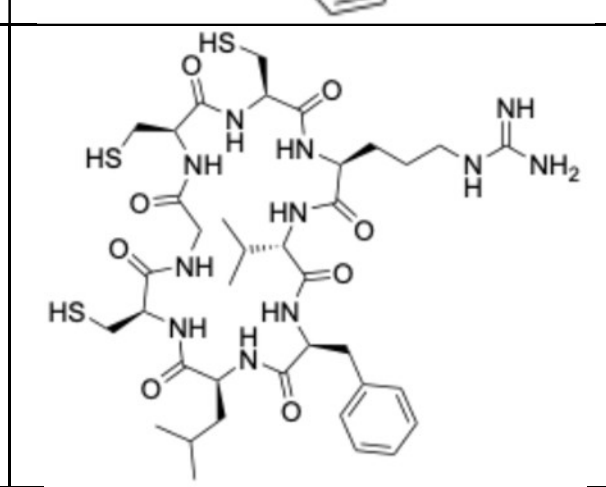	9x10^5 (apparent) and ~1x10^9 (theoretical)	*S. aureus* β-sliding clamp	R2H	Inhibited *S. aureus* growth with an MIC of ~50 μM	Suggested to be permeable to *S. aureus* cells

[15]	Head-to-tail CP	*E. coli*	CXXXXX	CLRFT* (* = stop codon)	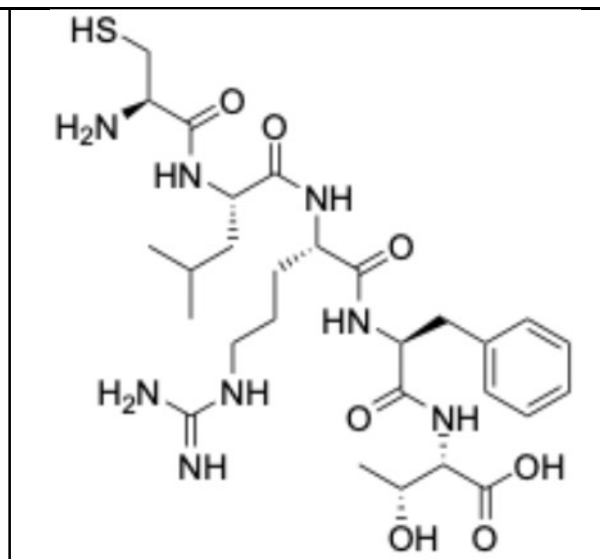	3x10^7 (apparent) and ~3.3x10^7 (theoretical)	*B. anthracis* protective antigen:human CMG2 receptor interaction	R2H	Bound CMG2 with a K_d of ~30 μM	Permeable to mammalian cells (BHK-21 and J774). May localize to cell membrane.
[16]	Head-to-tail CP	*E. coli*	$NuX_1X_2X_n$ (Nu = C, S, or T) (n = 3, 4, 5)	TAFDR	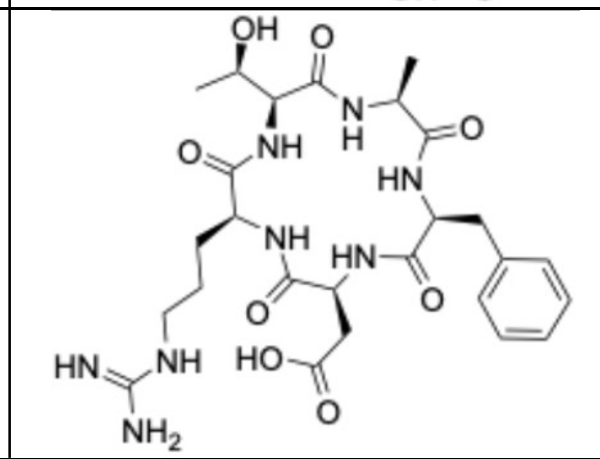	~1x10^8 (apparent) and ~3.5x10^7 (theoretical)	Amyloid-β 42 (Aβ42)	APP-GFP reporter	Several *in vitro* assays validated inhibitory activity on Aβ42 aggregation, but quantifiable potency is unclear.	Not tested
[16]	Head-to-tail CP	*E. coli*	$NuX_1X_2X_n$ (Nu = C, S, or T) (n = 3, 4, 5)	TWSVW	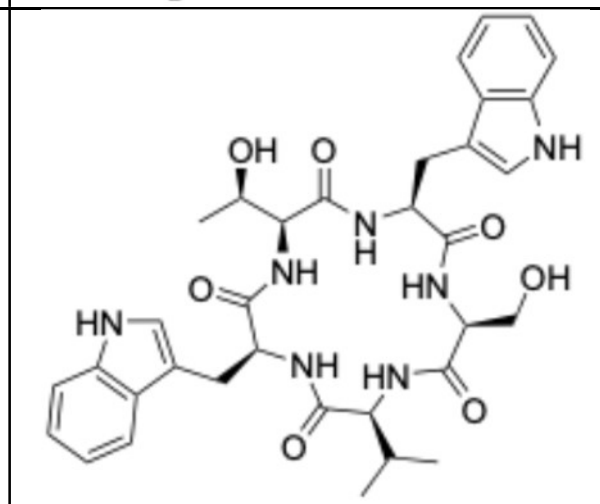	~1x10^8 (apparent) and ~3.5x10^7 (theoretical)	Mutant human Cu/Zn superoxide dismutase (SOD1 A4V)	APP-GFP reporter	Several *in vitro* assays validated inhibitory activity on SOD1 A4V aggregation, but quantifiable potency is unclear.	Not tested
[17]	Head-to-tail CP	*E. coli*	CXXXXX	CFFLYT	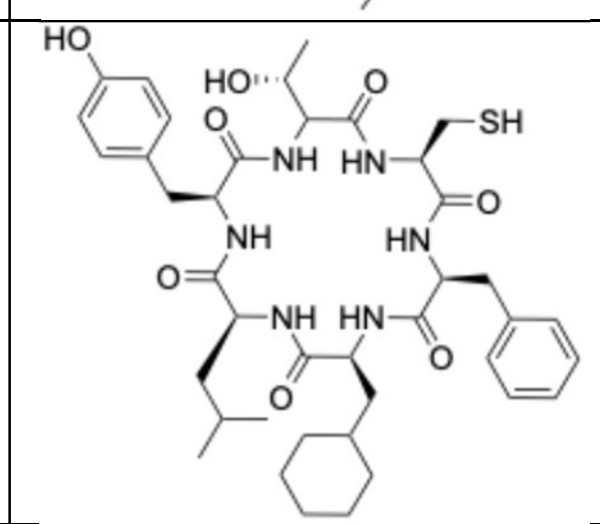	~7x10^6 (apparent) and ~3.3x10^7 (theoretical)	E3 ubiquitin ligase IDOL	R2H	Bound E3 ubiquitin ligase IDOL with a K_d of ~5 μM.	Permeable to mammalian cells (HepG2) when fused to Tat peptide
[18]	Lanthipeptide	*E. coli*	Leader-$AACX_5SMPPSX_5C$ (X = D, F, H, I, L, N, V, Y)	Leader-AACLHFFLSMPPSHVLDIC	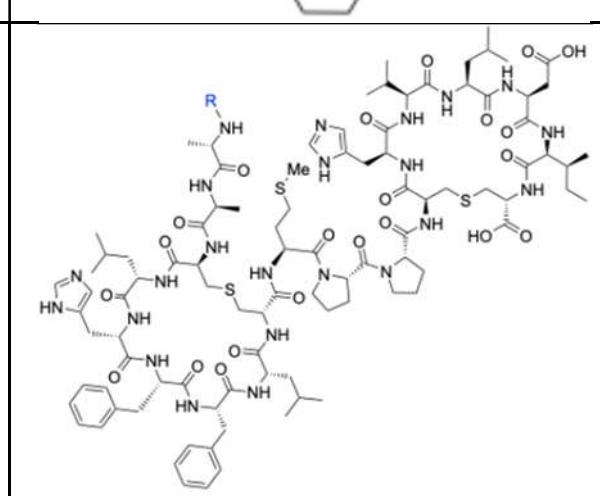 (R = GVAGG)	~1x10^6 (apparent) and 1.07x10^9 (theoretical)	p6:UEV interaction	R2H	Bound UEV with a Kd of ~4 μM and inhibited p6:UEV interaction with an IC_{50} of 1.9 μM.	Permeable to mammalian cells (293T) when fused to Tat peptide (N-terminal glycine replaced with cysteine for conjugation)

[19]	Head-to-tail CP	*E. coli*	CXXXXX	CIYYCV	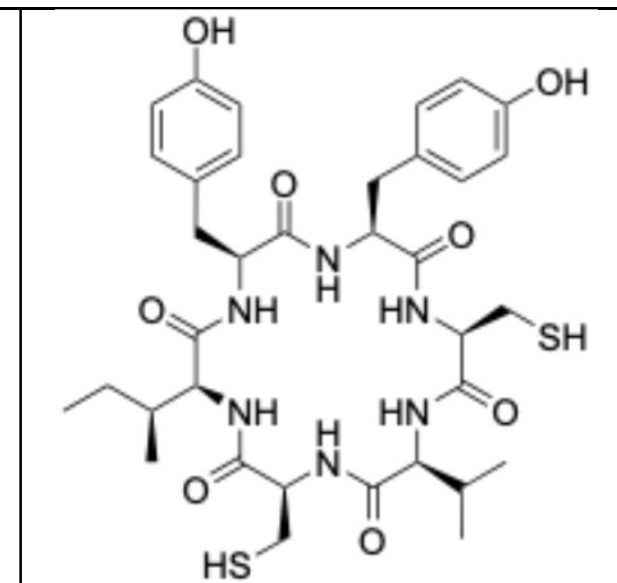	~1x10^7 (apparent) and 3.3x10^7 (theoretical)	Lymphoma 6 transcription factor's bric-a-brac, tramtrack and broad-complex (BTB) domain	R2H	Bound BTB with a K_d of ~50 µM.	Not tested
[20]	Head-to-tail CP	*E. coli*	NuXXXXXX (Nu = C, S, T)	CKVWQLL	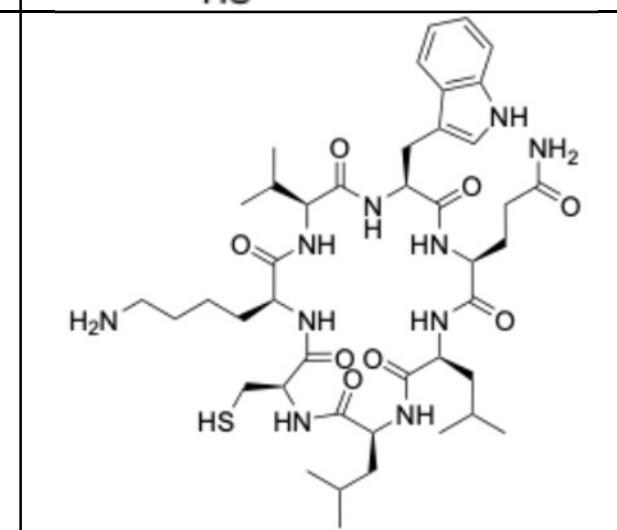	1.2x10^9 (apparent) and ~3x10^9 (theoretical)	Aβ42	APP-GFP reporter	Several *in vitro* assays validated inhibitory activity on Aβ42 aggregation, but quantifiable potency is unclear.	Not tested
[21]	Ranthipeptide	*E. coli*	Leader-XCX_3(D/E)XCX_3(D/E)X	Leader-VCKYGEWCEIVEI	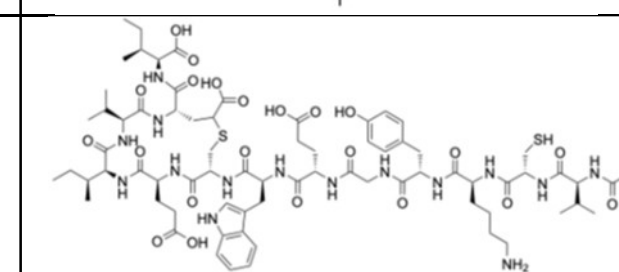	~1x10^8 (apparent) and ~1x10^{12} (theoretical)	SARS-CoV-2 spike protein	See the reference	Bound SARS-CoV-2 spike protein with a K_d of 0.99 µM.	Not tested
[22]	SICLOPPS lariat peptide	*S. cerevisiae*	SXXXXXXXX-Ic	SRYFFDMWY-Ic	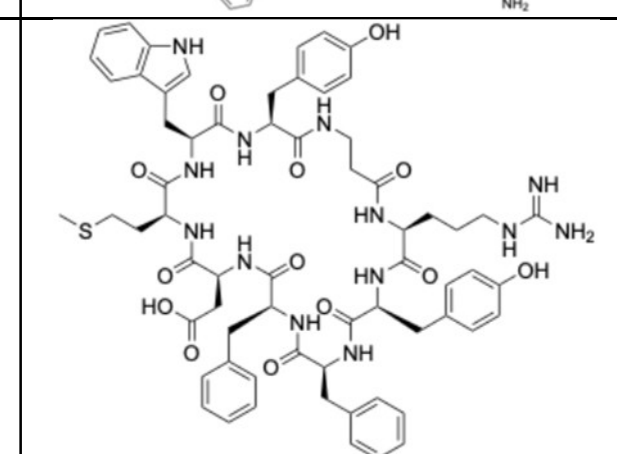	~5x10^6 (apparent) and ~1x10^{12} (theoretical)	GAL4 yeast transcription factor	Y2H	Exogenous addition of chemically re-synthesized showed inhibitory activity in Y2H, but quantifiable potency is unclear.	Membrane permeable (PAMPA-BBB assay)
[23]	Head-to-tail CP	*E. coli*	CXXXXX	CRVLAA	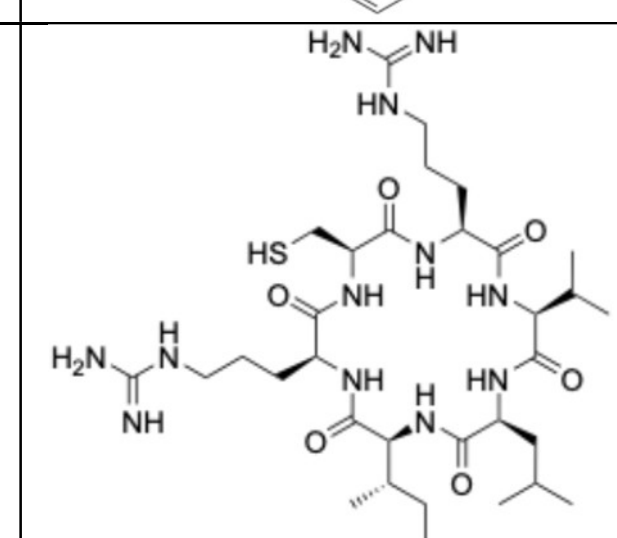	1x10^{10} (apparent) and ~3.3x10^7 (theoretical)	RBD of p110α:KRAS G12D interaction	R2H	Bound p110α with a K_d of 2.9 µM.	Permeable to mammalian cells (H1792)
[24]	SICLOPPS lariat peptide	*S. cerevisiae*	SXXX*XXXEY-Ic (* = 2-amino-8-oxodecanoic acid; X = any canonical amino acid and 2-amino-8-	SGGL*LWSEY-Ic (* = 2-amino-8-oxodecanoic acid)	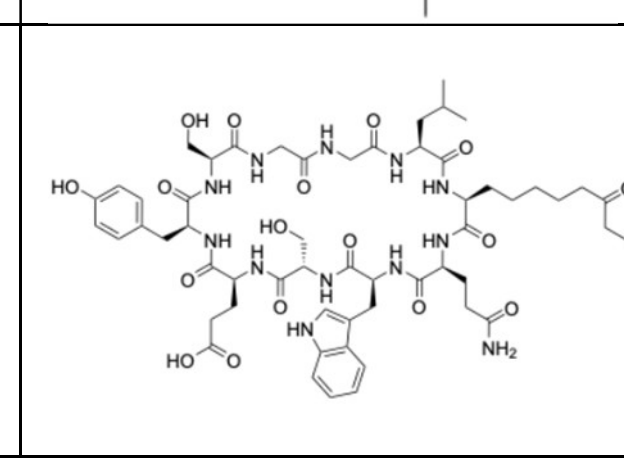	1.1x10^6 (apparent) and ~1x10^9 (theoretical)	Human histone deacetylase 8	Y2H	Bound human histone deacetylase 8 with a K_d of 15.1 nM and inhibited human histone deacetylase 8 with an IC_{50} of 0.61 µM.	Not tested

			oxodecanoic acid)							
[25]	Head-to-tail CP	*E. coli*	CXXXXX	CRLIIF	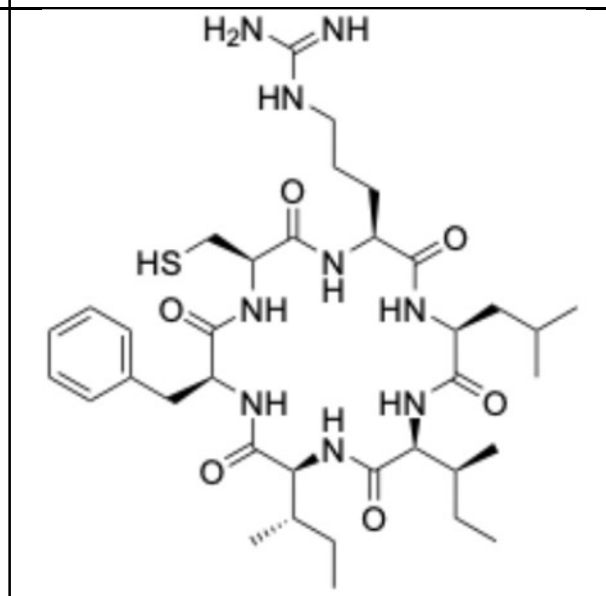	6.2×10^7 (apparent) and $\sim3.3\times10^7$ (theoretical)	HIF-1α:HIF-1β and HIF-2α:HIF-1β interactions	R2H	Bound HIF-1α with a K_d of 3.8 μM and HIF-2α with a K_d of 10.9 μM.	Derivative CRLII(4-I-F) found permeable to mammalian cells (T-REx-293)
[26]	Head-to-tail CP	*E. coli*	CXXXXX	CLLFCL	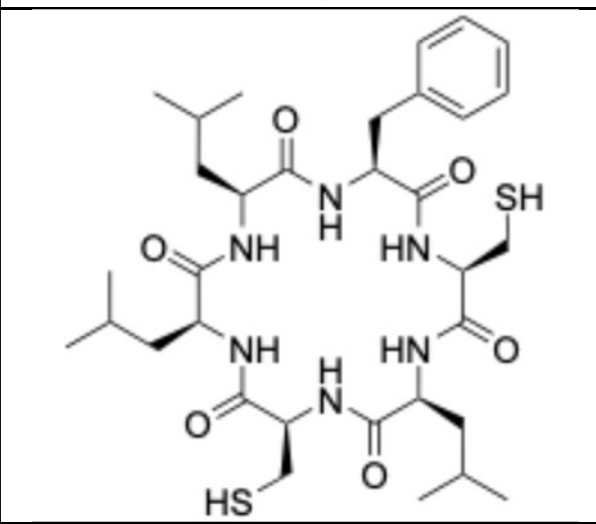	$\sim3.3\times10^7$ (theoretical)	HIF-1α:HIF-1β interaction	R2H	Bound HIF-1α with a K_d of 0.47 μM and inhibited HIF-1 activity in mammalian cells with an IC_{50} of 4.4 μM.	Permeable to mammalian cells (U2OS and MCF-7) when fused to Tat peptide
[27]	Head-to-tail CP	*E. coli*	CXXXXXX (X = F, L, I, V, T, S, Y, H, N, D, C, R)	CRCVFSG	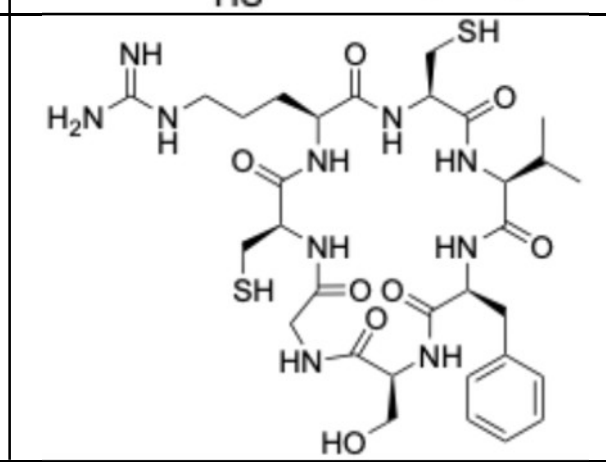	5×10^6 (apparent) and $\sim3\times10^6$ (theoretical)	Human islet amyloid poplypeptide (hIAPP)	APP-split T7 RNA polymerase reporter	Several *in vitro* assays validated inhibitory activity on hIAPP aggregation, but quantifiable potency is unclear.	Not tested
[28]	Head-to-tail CP	*E. coli*	CXXXXXX	CRLISFF	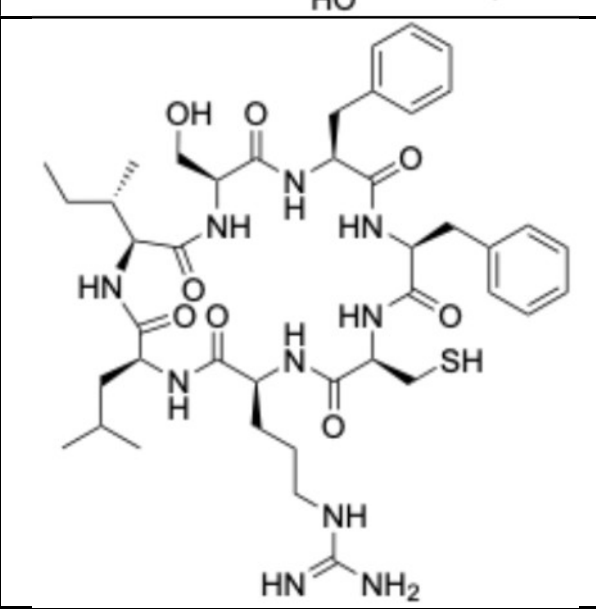	$\sim1\times10^7$ (apparent) and $\sim1\times10^9$ (theoretical)	Aβ42	cCadC-Aβ42 reporter	Inhibited Aβ42 aggregation in ThT fluorescence assay, but quantifiable potency is unclear.	Not tested

Supplementary Table 1. Summary of studies on cellular selection of genetically encoded cyclic peptide libraries to identify inhibitors for therapeutic targets.

Note: [a]: X = any canonical amino acid unless specified. [b]: In some cases, the peptide synthesized and tested *in vitro/in cellulo* is not the same as the peptide sequence enriched after selection due to sequence optimization, requirement of permeability tag, etc. Here, we prioritize showing the peptide tested *in vitro* that has the highest potency and cell permeability. [c]: Only one representative sequence is shown. The choice of this representative sequence is based on enrichment after selection and/or activity when expressed and tested in host cells. [d]: Apparent library size is defined as the number of transformants obtained from library generation. In cases where this information is unavailable, apparent library size refers to the number of transformants used for selection. [e]: Theoretical library size is defined as the number of all possible unique DNA

sequences encoded by the library. [f]: We direct readers to the corresponding reference when the selection system is a customized genetic circuit or does not belong to any of the generalizable selection systems we discussed in the review.

Supplementary References

1. Kinsella TM, Ohashi CT, Harder AG, et al (2002) Retrovirally delivered random cyclic peptide libraries yield inhibitors of interleukin-4 signaling in human B cells. Journal of Biological Chemistry 277:37512–37518. https://doi.org/10.1074/jbc.M206162200

2. Horswill AR, Savinov SN, Benkovic SJ (2004) A systematic method for identifying small-molecule modulators of protein-protein interactions. PNAS 101:15591–15596

3. Tavassoli A, Benkovic SJ (2005) Genetically selected cyclic-peptide inhibitors of AICAR transformylase homodimerization. Angew Chem Int Ed 44:2760–2763. https://doi.org/10.1002/anie.200500417

4. Tavassoli A, Lu Q, Gam J, et al (2008) Inhibition of HIV budding by a genetically selected cyclic peptide targeting the Gag-TSG101 interaction. ACS Chem Biol 3:757–764. https://doi.org/10.1021/cb800193n

5. Naumann TA, Tavassoli A, Benkovic SJ (2008) Genetic selection of cyclic peptide dam methyltransferase inhibitors. ChemBioChem 9:194–197. https://doi.org/10.1002/cbic.200700561

6. Cheng L, Naumann TA, Horswill AR, et al (2007) Discovery of antibacterial cyclic peptides that inhibit the ClpXP protease. Protein Science 16:1535–1542. https://doi.org/10.1110/ps.072933007

7. Kritzer JA, Hamamichi S, McCaffery JM, et al (2009) Rapid selection of cyclic peptides that reduce α-synuclein toxicity in yeast and animal models. Nat Chem Biol 5:655–663. https://doi.org/10.1038/nchembio.193

8. Barreto K, Bharathikumar VM, Ricardo A, et al (2009) A Genetic Screen for Isolating “Lariat” Peptide Inhibitors of Protein Function. Chem Biol 16:1148–1157. https://doi.org/10.1016/j.chembiol.2009.10.012

9. Young TS, Young DD, Ahmad I, et al (2011) Evolution of cyclic peptide protease inhibitors. PNAS 108:11052–11056. https://doi.org/10.1073/pnas.1108045108/-/DCSupplemental

10. Birts CN, Nijjar SK, Mardle CA, et al (2013) A cyclic peptide inhibitor of C-terminal binding protein dimerization links metabolism with mitotic fidelity in breast cancer cells. Chem Sci 4:3046–3057. https://doi.org/10.1039/c3sc50481f

11. Miranda E, Nordgren IK, Male AL, et al (2013) A cyclic peptide inhibitor of HIF-1 heterodimerization that inhibits hypoxia signaling in cancer cells. J Am Chem Soc 135:10418–10425. https://doi.org/10.1021/ja402993u

12. El-Mowafi SA, Alumasa JN, Ades SE, Keiler KC (2014) Cell-based assay to identify inhibitors of the Hfq-sRNA regulatory pathway. Antimicrob Agents Chemother 58:5500–5509. https://doi.org/10.1128/AAC.03311-14

13. El-Mowafi SA, Sineva E, Alumasa JN, et al (2015) Identification of inhibitors of a bacterial sigma factor using a new high-throughput screening assay. Antimicrob Agents Chemother 59:193–205. https://doi.org/10.1128/AAC.03979-14

14. Kjelstrup S, Hansen PMP, Thomsen LE, et al (2013) Cyclic Peptide Inhibitors of the β-Sliding Clamp in Staphylococcus aureus. PLoS One 8:e72273. https://doi.org/10.1371/journal.pone.0072273

15. Male AL, Forafonov F, Cuda F, et al (2017) Targeting Bacillus anthracis toxicity with a genetically selected inhibitor of the PA/CMG2 protein-protein interaction. Sci Rep 7:3104. https://doi.org/10.1038/s41598-017-03253-3

16. Matis I, Delivoria DC, Mavroidi B, et al (2017) An integrated bacterial system for the discovery of chemical rescuers of disease-associated protein misfolding. Nat Biomed Eng 1:838–852. https://doi.org/10.1038/s41551-017-0144-3

17. Leitch EK, Elumalai N, Fridén-Saxin M, et al (2018) Inhibition of low-density lipoprotein receptor degradation with a cyclic peptide that disrupts the homodimerization of IDOL E3 ubiquitin ligase. Chem Sci 9:5957–5966. https://doi.org/10.1039/c8sc01186a

18. Yang X, Lennard KR, He C, et al (2018) A lanthipeptide library used to identify a protein-protein interaction inhibitor article. Nat Chem Biol 14:375–380. https://doi.org/10.1038/s41589-018-0008-5

19. Osher EL, Castillo F, Elumalai N, et al (2018) A genetically selected cyclic peptide inhibitor of BCL6 homodimerization. Bioorg Med Chem 26:3034–3038. https://doi.org/10.1016/j.bmc.2018.03.012

20. Delivoria DC, Chia S, Habchi J, et al (2019) Bacterial production and direct functional screening of expanded molecular libraries for discovering inhibitors of protein aggregation. Sci Adv 5:. https://doi.org/10.1126/sciadv.aax5108

21. King AM, Anderson DA, Glassey E, et al (2021) Selection for constrained peptides that bind to a single target protein. Nat Commun 12:. https://doi.org/10.1038/s41467-021-26350-4

22. Rosa S, Tagliani A, Bertaso C, et al (2023) The cyclic peptide G4CP2 enables the modulation of galactose metabolism in yeast by interfering with GAL4 transcriptional activity. Front Mol Biosci 10:. https://doi.org/10.3389/fmolb.2023.1017757

23. Ismail M, Martin SR, George R, et al (2023) Characterisation of a cyclic peptide that binds to the RAS binding domain of phosphoinositide 3-kinase p110α. Sci Rep 13:1889. https://doi.org/10.1038/s41598-023-28756-0

24. Kang D, Kim DW, Kim JC, Park HS (2023) A Versatile Strategy for Screening Custom-Designed Warhead-Armed Cyclic Peptide Inhibitors. Angew Chem Int Ed 62:4–9. https://doi.org/10.1002/anie.202214815

25. Ball AT, Mohammed S, Doigneaux C, et al (2024) Identification and Development of Cyclic Peptide Inhibitors of Hypoxia Inducible Factors 1 and 2 That Disrupt Hypoxia-Response Signaling in Cancer Cells. J Am Chem Soc 146:8877–8886. https://doi.org/10.1021/jacs.3c10508

26. McDermott A, Windeln LM, Valentine JSD, et al (2024) Next Generation SICLOPPS Screening for the Identification of Inhibitors of the HIF-1α/HIF-1β Protein-Protein Interaction. ACS Chem Biol 19:2232–2239. https://doi.org/10.1021/acschembio.4c00494

27. Yang L, Zhang J, Andon JS, et al (2025) Rapid discovery of cyclic peptide protein aggregation inhibitors by continuous selection. Nat Chem Biol 21:588–597. https://doi.org/10.1038/s41589-024-01823-x

28. Lee BU, Flood B, Potter E, Wang T (2026) Identification of Secondary Nucleation Inhibitors of Amyloid-β Aggregation by Cellular Selection of a SICLOPPS Library. ChemBioChem 27:e202500908. https://doi.org/10.1002/cbic.202500908
